# Supplementary material for: Evaluating practical support stroke survivors get with medicines and unmet needs in primary care: a survey
Source: BMJ Open. 2018 Mar 11;8(3):e019874. doi: 10.1136/bmjopen-2017-019874 (PMC5855212; doi:10.1136/bmjopen-2017-019874)
Supplement: Supplementary data [file bmjopen-2017-019874supp001.pdf]

Queen Mary University of London

UNIVERSITY OF CAMBRIDGE

QUESTIONNAIRE – PATIENT

Many people with stroke need help with taking their medicines. We would like to ask you few questions about the help you might need with taking your medicines.

How many different type of medicines do you take in one day? ☐
Write the number or an approximation. Please count all medications including eye drops, injections etc.

How old are you? ☐ How long ago was your stroke? ☐ years

What is your sex? M ☐ F ☐

For each question below, please tick the box that best describes how you have taken your medicines <u>in the last month</u>:

1. **Is somebody helping with prescriptions and collection of your medicines?**

   all the time ☐
   often ☐
   sometimes ☐
   rarely ☐
   never ☐

   Do you feel you need more help with prescriptions and collection of your medicines?

   Yes ☐ **No** ☐

2. **Is somebody helping you getting the medicines out of the box, bottle or blister pack?**

   all the time ☐
   often ☐
   sometimes ☐
   rarely ☐
   never ☐

   Do you feel you need more help with getting the medicines out of the box, bottle or blister pack?

   Yes ☐ **No** ☐

3. Is somebody helping with reminding you when is the time to take your medicine?

all the time ☐
often ☐
sometimes ☐
rarely ☐
never ☐

Do you feel you need more help with reminding when is the time to take your medicine?

Yes ☐ **No** ☐

4. Is somebody helping you with swallowing your medicine? For example by giving you a drink

all the time ☐
often ☐
sometimes ☐
rarely ☐
never ☐

Do you feel you need more help with swallowing your medicine?

Yes ☐ **No** ☐

5. Is somebody helping you with checking that you have taken your medicines?

all the time ☐
often ☐
sometimes ☐
rarely ☐
never ☐

Do you feel you need more help with checking that you have taken your medicine?

Yes ☐ **No** ☐

Missing medicines

Thinking of the last 30 days, how often did you miss taking your regular medicines?

Remember - tick one box only

all the time ☐
often ☐
sometimes ☐
rarely ☐
never ☐

Barthel Questionnaire

These are some questions about your ability to look after yourself.

They may not seem to apply to you.

Please answer them all.

Tick one box in each section.

Bathing

In the bath or shower do you:	manage on your own?	☐
	need help getting in and out?	☐
Remember - tick one box only	need other help?	☐
	never have a bath or shower?	☐
	need to be washed in bed?	☐

Stairs

Do you climb stairs at home:	without any help?	☐
	with someone carrying your frame?	☐
Remember - tick one box only	with someone encouraging you?	☐
	with physical help?	☐
	not at all?	☐
	don’t have stairs?	☐

Dressing

Do you get dressed:	without any help?	☐
	just with help with buttons?	☐
Remember - tick one box only	with someone helping you most of the time?	☐

Mobility

Do you walk indoors:	without any help apart from a frame?	☐
	with one person watching over you?	☐
Remember - tick one box only	with one person helping you?	☐
	with more than one person helping?	☐
	not at all?	☐
	Or do you use a wheelchair independently? (e.g. round corners)	☐

Transfer

Do you move from bed to chair:	on your own?	☐
	with a little help from one person?	☐
Remember - tick one box only	with a lot of help from one or more people?	☐
	not at all?	☐

Feeding

Do you eat food:		
	without any help?	☐
	with help cutting food or spreading butter?	☐
Remember - tick one box only	with more help?	☐

Toilet use

Do you use a toilet or commode:		
	without any help?	☐
	with some help but can do something?	☐
Remember - tick one box only	with quite a lot of help?	☐

Grooming

Do you brush your hair and teeth Wash your face and shave:		
	without help?	☐
Remember - tick one box only	with help?	☐

Bladder

Are you incontinent of urine?		
	never	☐
	less than once a week	☐
Remember - tick one box only	less than once a day	☐
	more often	☐
	Or do you have a catheter managed for you	☐

Bowels

Do you soil yourself?		
	never	☐
	Occasional accident	☐
Remember - tick one box only	all the time	☐
	or do you need someone to give you an enema?	☐
