# Supplementary material for: Evaluating practical support stroke survivors get with medicines and unmet needs in primary care: a survey
Source: BMJ Open. 2018 Mar 11;8(3):e019874. doi: 10.1136/bmjopen-2017-019874 (PMC5855212; doi:10.1136/bmjopen-2017-019874)
Supplement: Supplementary data [file bmjopen-2017-019874supp002.pdf]

		.			

Queen Mary University of London

UNIVERSITY OF CAMBRIDGE

Help with taking tablets after stroke

FAMILY MEMBER/FRIEND OR PRIVATE CARER COPY

Many people with stroke need help with taking their medicines. We would like to ask you few questions about the help you might be offering to your family member/friend/ patient with stroke with taking medicines.

Relation with your family member/friend with stroke

Are you:

Remember - tick one box only

- partner ☐
- son or daughter ☐
- friend ☐
- carer from an agency ☐
- other ☐
- if other, please specify

How many different types of medicines does your family member/friend/patient with stroke take in one day? ☐
Write the number or an approximation. Please count all medications including eye drops, injections etc.

How old is your family member/friend/patient with stroke? (years) ☐

How many years ago was your family member/friend/patient's stroke? ☐

What is your family member/friend/patient with stroke sex? M ☐ F ☐

For each question below, please tick the box that best describes the help needed by your family member/friend/patient with stroke with taking medicines <u>in the last month.</u>

1. **Is somebody helping your family member/friend/patient with stroke with prescriptions and collection of his/her medicines?**
   - all the time ☐
   - often ☐
   - sometimes ☐
   - rarely ☐
   - never ☐

   Do you feel your family member/friend/patient with stroke needs more help with prescriptions and collection of his/her medicines?

   Yes ☐ **No** ☐

2. **Is somebody helping your family member/friend/patient with stroke getting the medicines out of the box, bottle or blister pack?**

- all the time ☐
- often ☐
- sometimes ☐
- rarely ☐
- never ☐

Do you feel your family member/friend/patient with stroke needs more help with getting the medicines out of the box, bottle, or blister pack?

Yes ☐ **No** ☐

3. **Is somebody helping your family member/friend/patient with stroke with reminding when is time to take his/her medicine?**

- all the time ☐
- often ☐
- sometimes ☐
- rarely ☐
- never ☐

Do you feel your family member/friend/patient with stroke needs more help with reminding when is time to take his/her medicine?

Yes ☐ **No** ☐

4. **Is somebody helping your family member/friend/patient with stroke with swallowing his/her medicine? For example by giving a drink.**

- all the time ☐
- often ☐
- sometimes ☐
- rarely ☐
- never ☐

Do you feel you your family member/friend/patient with stroke need more help with swallowing his/her medicine?

Yes ☐ **No** ☐

5. **Is somebody helping your family member/friend/patient with stroke with checking that he/she has taken his/her medicines?**

- all the time ☐
- often ☐
- sometimes ☐
- rarely ☐
- never ☐

Do you feel your family member/friend/patient with stroke needs more help with checking that he/she has taken his/her medicine?

Yes ☐ **No** ☐

☐ ☐ . ☐ ☐ ☐

Missing medicines

Thinking of the last 30 days, how often did your family member/friend/patient with stroke miss taking his/her regular medicines?	all the time	☐
	often	☐
	sometimes	☐
Remember - tick one box only	rarely	☐
	never	☐

Barthel Questionnaire

These are some questions about the ability of your family member/friend/patient with stroke to look after him/herself.

Please answer them all.

Please fill this questionnaire even if you are not regularly caring for your family member/friend/patient with stroke, trying to answer questions in the way you think most accurately describes the disability of your family member/friend/patient with stroke.

Tick one box in each section.

Bathing

In the bath or shower do you:	manage on your own?	☐
	need help getting in and out?	☐
Remember - tick one box only	need other help?	☐
	never have a bath or shower?	☐
	need to be washed in bed?	☐

Stairs

Do you climb stairs at home:	without any help?	☐
	with someone carrying your frame?	☐
Remember - tick one box only	with someone encouraging you?	☐
	with physical help?	☐
	not at all?	☐
	don’t have stairs?	☐

Dressing

Do you get dressed:	without any help?	☐
	just with help with buttons?	☐
Remember - tick one box only	with someone helping you most of the time?	☐

Mobility

Do you walk indoors:	without any help apart from a frame?	☐
	with one person watching over you?	☐
Remember - tick one box only	with one person helping you?	☐

☐ ☐ . ☐ ☐ ☐

	with more than one person helping?	☐
	not at all?	☐
	Or do you use a wheelchair independently? (e.g. round corners)	☐

Transfer

Do you move from bed to chair:	on your own?	☐
	with a little help from one person?	☐
Remember - tick one box only	with a lot of help from one or more people?	☐
	not at all?	☐

Feeding

Do you eat food:	without any help?	☐
	with help cutting food or spreading butter?	☐
Remember - tick one box only	with more help?	☐

Toilet use

Do you use a toilet or commode:	without any help?	☐
	with some help but can do something?	☐
Remember - tick one box only	with quite a lot of help?	☐

Grooming

Do you brush your hair and teeth	without help?	☐
Wash your face and shave:	with help?	☐
Remember - tick one box only		

Bladder

Are you incontinent of urine?	never	☐
	less than once a week	☐
Remember - tick one box only	less than once a day	☐
	more often	☐
	Or do you have a catheter managed for you	☐

Bowels

Do you soil yourself?	never	☐
	Occasional accident	☐
Remember - tick one box only	all the time	☐
	or do you need someone to give you an enema?	☐
