# Supplementary material for: Evaluating practical support stroke survivors get with medicines and unmet needs in primary care: a survey
Source: BMJ Open. 2018 Mar 11;8(3):e019874. doi: 10.1136/bmjopen-2017-019874 (PMC5855212; doi:10.1136/bmjopen-2017-019874)
Supplement: Supplementary data [file bmjopen-2017-019874supp003.pdf]

	Unmet needs		Missing medication	
**Variable**	**N**	**Odds ratio (95% CI) p value**	**N**	**Odds ratio (95%CI) p value**
Age ≥70	498	0.7 (0.4-1.2) p=0.248	508	0.6 (0.4-0.9) p=0.009
Gender (female)	498	0.7 (0.4-1.3) p=0.262	508	0.9 (0.6-1.3) p=0.576
Number of different medicines	465	1.2 (1.1-1.3) p<0.001	475	1.1 (1.0-1.1) p=0.015
Moderate Dependence for ADLs (BI: 15-19)	408	2.6 (0.9-7.5) p<0.075	417	0.3 (-0.2-0.7) p=0.289
Severe Dependence for ADLs (BI: 0-14)	408	10.9 (3.8-31.0) p<0.001	417	0.5 (-0.1-1.0) p=0.119
Years since stroke	446	1.0 (1.0-1.1) p=0.036	454	1.0 (0.9-1.03) p=0.725
Getting help with prescriptions and collection of medication	487	4.6 (2.4-8.9) p<0.001	497	2.6 (1.8-3.8) p<0.001
Getting help with taking medicines out of the box, bottle or blister pack	481	6.6 (3.6-12.2) p<0.001	491	1.7 (1.1-2.6) p=0.20
Getting help with reminding you when is the time to take your medicine?	480	4.7 (2.6-8.5) p<0.001	490	3.0 (2.0-4.5) p<0.001
Getting help to swallow the medication	482	7.9 (4.2-14.8) p<0.001	492	2.1 (1.3-3.4) p=0.005
Getting help by checking that you have taken your medicines	480	5.9 (3.2-10.9) p<0.001	490	2.8 (1.8-4.2) p<0.001
Getting any kind of help	491	5.6 (2.7-11.9) p<0.001	501	2.3 (1.6-3.4) p<0.001

Supplementary Appendix 1. Predictors of unmet needs and missing medication, responses given by patients only (i.e. caregivers' filled questionnaire excluded). Multivariable analyses, all models adjusted for age and gender. N: number of observations.
