# Supplementary material for: Evaluating practical support stroke survivors get with medicines and unmet needs in primary care: a survey
Source: BMJ Open. 2018 Mar 11;8(3):e019874. doi: 10.1136/bmjopen-2017-019874 (PMC5855212; doi:10.1136/bmjopen-2017-019874)
Supplement: Author's manuscript [file bmjopen-2017-019874.draft_revisions.pdf]

BMJ Open

BMJ Open is committed to open peer review. As part of this commitment we make the peer review history of every article we publish publicly available.

When an article is published we post the peer reviewers' comments and the authors' responses online. We also post the versions of the paper that were used during peer review. These are the versions that the peer review comments apply to.

The versions of the paper that follow are the versions that were submitted during the peer review process. They are not the versions of record or the final published versions. They should not be cited or distributed as the published version of this manuscript.

BMJ Open is an open access journal and the full, final, typeset and author-corrected version of record of the manuscript is available on our site with no access controls, subscription charges or pay-per-view fees (http://bmjopen.bmj.com).

If you have any questions on BMJ Open's open peer review process please email info.bmjopen@bmj.com

BMJ Open

Evaluating support stroke survivors get with medicines and unmet needs in primary care: A survey

Journal:	*BMJ Open*
Manuscript ID	bmjopen-2017-019874
Article Type:	Research
Date Submitted by the Author:	02-Oct-2017
Complete List of Authors:	JAMISON, JAMES; UNIVERSITY OF CAMBRIDGE, PUBLIC HEALTH & PRIMARY CARE<br>Ayerbe, Luis; Queen Mary University of London, Centre for Primary Care and Public Health<br>Di Tanna, Gian Luca; Queen Mary University of London<br>Sutton, Stephen; University of Cambridge,<br>Mant, Jonathan; University of Cambridge, General Practice and Primary Care Research Unit<br>De Simoni, Anna; Queen Mary University of London, Centre for Primary Care and Public Health
Keywords:	Medication adherence, Caregivers, Barthel, STROKE MEDICINE

SCHOLARONE™ Manuscripts

For peer review only

Evaluating support stroke survivors get with medicines and unmet needs in primary care: A survey

*James Jamison[1], MSc

Luis Ayerbe[2], PhD

Gian Luca Di Tanna[2] PhD

Stephen Sutton[1], PhD

Jonathan Mant[1], MD

Anna De Simoni[2], PhD

[1]Primary Care Unit, Department of Public Health & Primary Care, Forvie Site, University of Cambridge School of Clinical Medicine, Box 113 Cambridge Biomedical Campus, Cambridge, CB2 0SR.

[2]Centre for Primary Care and Public Health, Barts and The London School of Medicine and Dentistry, Yvonne Carter Building, London E1 2AB.

*corresponding author: James Jamison; Tel: +44 (0)1223 768272. Fax: +44 (0)1223 763492. Email:

jj285@medschl.cam.ac.uk

For peer review only

Abstract

Objectives

To design a questionnaire and use it to explore unmet needs with practical aspects of medicines taking after stroke, predictors of medicine taking and to estimate the proportion of survivors who get support with daily medication taking.

Design

Four workshops with stroke survivors and caregivers to design the questionnaire.

A cross-sectional postal questionnaire in primary care.

Setting

18 GP practices in the East of England and London. Questionnaires posted between September 2016 and February 2017.

Participants

1687 stroke survivors living in the community outside institutional long term care.

Primary Outcome measures

The proportion of community stroke survivors receiving help from caregivers for practical aspects of medicine taking; the proportion with unmet needs in this respect; the predictors of experiencing unmet needs and missing taking medications.

Results

A 5-item questionnaire was developed to cover the different aspects of medicine taking. 596/1687 (35%) questionnaires were returned. 56% reported getting help in at least one aspect of taking medication and 11% needing more help. 33% reported missing taking their medicines. Unmet needs were associated with receiving help with medications (OR: 5.6, $p<0.001$), being on a higher number of medications (OR: 1.2, $p<0.001$) and being dependent for activities of daily living (ADL) (OR: 4.9, $p=0.001$). Missing medication was associated with having unmet needs (OR: 5.1, $p<0.001$), receiving help with medications (OR: 2.1, $p<0.001$), being on a higher number of medicines (OR: 1.1, $p=0.008$) and being older than 70 years (OR: 0.6, $p=0.006$).

For peer review only - http://bmjopen.bmj.com/site/about/guidelines.xhtml

Conclusions

More than half of patients who replied needed help with taking medication and 1 in 10 had unmet needs in this regard. Stroke survivors dependent on others have more unmet needs, more likely to miss medicines and might benefit from focused clinical and research attention. Novel primary care interventions focusing on the practicalities of taking medicines are warranted.

Abstract word count- 298

Keywords: Stroke, Medication Adherence, Caregivers, Barthel

Article summary

Strengths and Limitations

- A 5-item questionnaire was developed to evaluate the help stroke survivors get with daily medication taking, based on patients' and caregivers' own views gathered through workshops.
- The questionnaire was sent to 1,687 stroke survivors in 18 GP practices across two UK regions.
- This work identified issues from a population that includes patients severely affected by stroke, who are often excluded from research. Results shed light on the effect of stroke related impairments on practical domains and predictors of medicine taking, which have significant effects on overall adherence and call for new primary care interventions.
- The low response rate reported is a limitation of this study and stroke survivors who are harder to reach may have been missed.

Funding: This study was funded by the RCGP SFB, Ref. SFB 2014 – 15 'Quantifying the support stroke survivors get with daily medication taking: a questionnaire survey'. Anna De Simoni and Luis Ayerbe are funded by a NIHR Academic Clinical Lectureships. This article therefore presents independent research funded by NIHR. The views expressed are those of the authors and not necessarily those of the NHS, the NIHR, or the Department of Health. James Jamison was supported by a research grant from The Stroke Association and the British Heart Foundation: TSA BHF 2011/01

For peer review only

For peer review only - http://bmjopen.bmj.com/site/about/guidelines.xhtml

Data sharing statement: No additional data available.

Introduction

For many older adults remaining independent at home may depend on how well they can manage complex medication regimens.[1 2] Around half of stroke survivors are dependent on others for everyday activities.[3] Stroke is the leading cause of disability in developed countries, with an estimated that 25-74% of the 50 million stroke survivors worldwide requiring some assistance or being fully dependent on caregivers for activities of daily living (ADL's).[4-6]

There is evidence that being dependent for ADL's and impairment in mobility and communication decrease medication adherence in patients suffering from hypertension.[7] Deficits in attention, cognition or working memory have been linked with non-adherence to medications in other patient groups.[8] In a recent systematic review of medication adherence among patients with cognitive impairment, one third of studies showed that such patients were likely to have a caregiver to assist with medications and there was an association between taking four or more medicines and nonadherence.[9] In patients taking cardiovascular medicines, multiple factors including cognitive problems, lack of social support, dosing regimen, as well as practical problems and difficulties accessing services, contribute to poor medication adherence.[10 11] Low adherence to secondary prevention medication is associated with poor cardiovascular health.[12 13]

Stroke survivors have previously reported difficulties in the handing of medication as a barrier to adherence to secondary prevention medication after stroke.[14] Research on medication adherence in stroke has identified multiple barriers to medication taking among stroke survivors.[14-16] However interventions developed to improve adherence have mainly concentrated on patients responsible for their own medicine taking.[17 18]

In elderly patients in particular, cognitive deficits, taking large number of medicines and the complexity of medication regimens have been identified as barriers to medication adherence. [19 20] Caregivers are known to play a key role in providing assistance to older people in a range of daily activities including medication taking and physician visits,[21] and can help improve adherence in cardiac patients with memory

For peer review only - http://bmjopen.bmj.com/site/about/guidelines.xhtml

problems.[22] However, the proportion of community stroke survivors relying on caregivers for some, or all aspects of medicine taking, is not known.

Survivors of stroke have previously reported unmet needs including physical difficulties, cognitive and emotional difficulties, information needs and other unmet needs.[23 24] However we know little about factors that influence medication taking among stroke survivors with disabilities living in the community (i.e. not in nursing homes), their unmet needs around the use of medicines or the proportion relying on caregivers for some or all aspects of medicines taking.

To date, survey instruments examining the unmet needs of stroke survivors have not focused on aspects of medication taking.

The aims of this investigation were to design an instrument to evaluate the help stroke survivors get with taking their medicines, characterise patients receiving help with medications, estimate the proportion who have unmet needs with daily medicine taking and who miss medications. We additionally aimed to identify the predictors of missing medicines and of experiencing unmet needs with medications.

This knowledge can inform the development of primary care interventions aimed at improving medication taking in this patients' group.

Methods

Questionnaire development workshops

To develop the questionnaire, three workshops were conducted with 26 stroke survivors and 12 caregivers in the East of England (St John's College, Cambridge 2009 [25]: 7 patients, 1 caregiver; Different Stroke, Cambridge 2012: 9 patients, 3 caregivers; Peterborough, 2012: 10 patients, 8 caregivers). Recruitment was opportunistic and no purposive sampling was applied.

The survey questions were developed through thematic analysis [26] of workshops field notes.

A fourth workshop was conducted to gather feedback on the questionnaire using a PPI (Patient and Public Involvement) exercise with 11 stroke survivors and 3 caregivers recruited through a local stroke group (Different Strokes, East of England). Two stroke survivors from this group took part in subsequent 'think-

For peer review only

aloud' interviews, which involved talking out loud as they read the questionnaire, continually verbalising what they were thinking.

Postal survey

General practices in primary care in the East of England and London were approached through the Clinical Research Network (CRN). CRN Eastern contacted 20 GP practices, of which 11 replied and took part in the study. CRN North London contacted 140 GP East London practices by email (Tower Hamlets, Newham and City & Hackney CCGs), of which only two replied and participated in the study. Five of the eight GP practices contacted in North London (Barnet CCG) through a research coordinator took part in the study. Patients with stroke and their caregivers were sent the postal questionnaire according to the following criteria

Inclusion Criteria

Patients:

All patients aged > 18 on the practice stroke register with documented history of stroke.

Caregivers:

- Anyone identified by the patient as being a caregiver and having a role helping with medicine taking.

Exclusion Criteria

- Patients who suffered a Transient ischaemic attack (TIA) but not a stroke.
- Palliative or end of life patients.
- Patients receiving institutional long term care (receiving total care in residential homes or living in nursing homes).

Survey participant identification

A list of prospective patients was compiled from the stroke register of each surgery by the practice staff. No restriction was placed on the recruitment of survivors experiencing who were dependent for ADL's or lacking capacity. The list was screened by a practice GP and anyone not meeting the inclusion criteria or whom the GP considered unsuitable for the study (e.g. terminally ill) was excluded.

Survey participant recruitment

For peer review only - http://bmjopen.bmj.com/site/about/guidelines.xhtml

Eligible participants were sent a study survey pack by practice staff between September 2016 and February 2017. Study recruitment packs included two invitation letters, patient information sheets, questionnaires and postal version of Barthel Index[27], one of which was for completion by the patient and the other by the caregiver. The Barthel Index provides a measure of functional independence and physical functioning and has been used in stroke research previously.[28] Patients with Barthel score <20 were categorised as dependent for ADLs/disabled. If receiving help with medications, the patient was asked to pass to their caregiver the invitation letter and information sheet and invite him/her to complete their copy of the questionnaire, providing answers on the patient's medicine taking. Family members, friends or paid caregivers of stroke survivors who were severely disabled and/or lacked mental capacity were invited to fill and return the caregivers' questionnaires only on behalf of patients. The information sheets stated that consent was implied by returning the completed questionnaire. Participants were asked to return completed questionnaires to the research centre in the FREEPOST envelopes provided. A second mail out of the study invitation pack was sent to all patients as a reminder, 2 weeks after the first one.

Ethical approval

This study has received ethical approval from Cambridge Central Research Ethics Committee (REC reference: 16/EE/0182) and from the Health Research Authority (IRAS project ID:170931)

Survey Analysis

Survey data entry was performed by Document Capture Company.[29] Individual patients' characteristics (age, gender, time since stoke, number of daily medicines) were collected from the questionnaires themselves. Practice population, number of patients on stroke registers, deprivation score and ethnicity were taken from the National General Practice profiles (https://fingertips.phe.org.uk/profile/general-practice). The proportions of patients in each sociodemographic category, needing help taking medication, missing any medication in the previous 30 days, and reporting the need for more help taking medication, were estimated. When the survivor and caregiver questionnaires were both returned together, study data were collected from the patient's questionnaire only.

For peer review only - http://bmjopen.bmj.com/site/about/guidelines.xhtml

Multivariable logistic regression analysis was fitted to estimate associations between ‘Unmet needs’ and the variables: age < or ≥70 years, gender, total number of medicines taken, dependence for ADL, years since stroke, and receiving help with medicines. In the model each domain of help with medicine was estimated individually and then combined. A second multivariable logistic regression examined the association between ‘Missed medicines in the previous 30 days’ and the variables: age < or > 70 years, gender, total number of medicines taken, dependent for ADL, years since stroke, help with medicines and unmet needs. Regression models were adjusted for age, gender and variable of interest. All statistical analysis have been conducted with Stata (version 14, StataCorp LP, College Station, TX, USA, 2013).

Results

Questionnaire development

Taking medications emerged as an important issue in all three workshops: nearly half of patients stated that a family member or friend was supporting them with daily medicine routines especially in relation to prompting medicine taking. This was put down to effects of the stroke itself on memory retention rather than general memory problems that people without stroke also experience. They admitted missing doses due to forgetting. Only a small proportion of survivors were actually handling their own prescriptions and were relying on support from family and/or community services. In one workshop almost all survivors had Dosette medication boxes and agreed that taking medications out of safety bottles and blister packs was a problem due to physical disabilities.

Thematic analysis of workshop data revealed five main practical domains of support needed with medication taking: 1) Dealing with prescriptions and collection of medicines; 2) Getting medicines out of the box, blister packs of bottles; 3) Prompting ‘It’s time to take your medicine’; 4) Swallowing medicines; and 5) Checking whether medicines have been taken. The final study questionnaire (see Supplementary file 1) included questions relating to each of these five domains, one item related to adherence (missed medicine in the last 30 days) and an assessment of disabilities through completion of the validated postal version of the Barthel Index.[27] The questionnaire was adapted for caregivers (see Supplementary file 2).

For peer review only - http://bmjopen.bmj.com/site/about/guidelines.xhtml

Questionnaire finalisation

On the basis of the fourth workshop and two 'think-aloud' interviews, we reworded the survey questions (e.g. from ‘Do you get help with’ was changed into ‘Is somebody helping you with’) and used a scale response 'All the time', ‘Often’, ‘Sometimes’, ‘Rarely’, ‘Never’ for the first question of each of the five survey domains, which was originally conceived as a ‘yes’ or ‘no’ answer (see table 2 for text of questions).

Survey

Practice characteristics

18 GP practices agreed to take part in the study, of which just over 1/3 were in London (n=7). GP practices were relatively large with an average population of 11,904 patients (SD = 4010) and a low to moderate level of deprivation (Index of Multiple Deprivation[30] (IMD): Mean-7.05: SD-3.19). Out of 3066 patients on the stroke registers, 1687 stroke patients (55%) were considered eligible for the study and received the postal questionnaire. The average response rate of East of England and London practices was 42% and 27% respectively. The response rate varied between 16% and 53% across practices.

Participant characteristics

596 participants returned a completed questionnaire [549 (92.4%) from patients, 45 (7.6%) from caregivers] showing a mean response rate of 35% (0.33-0.37). Participants were on average 72.7yrs old. 37.8% (n=210) of the sample were female and 62.2% male (n=346), see table 1. The mean number of years since stroke was 7.7 and participants took an average of 6 different medicines a day. There were a high proportion of white patients in the recruited practices which were on average 21% of mixed or ethnic minority background. Approximately 23% of study participants were completely independent for ADL.

Participants getting any kind of help with medicines were on average 73.6 years old, two thirds were male, had a stroke approximately 8 years previously and were taking on average 1 extra medication a day. Only 19% of this group were completely independent for ADL, see second part of table 1.

Table 1 here

For peer review only - http://bmjopen.bmj.com/site/about/guidelines.xhtml

Support with daily medication taking

Table 2 shows the mean responses to the survey questions quantifying the help participants receive with medicines and unmet needs. Overall, 55.7% (95% CI: 51.7-59.7) of the participants received help in at least one aspect of taking medication, in that they ticked one of the options from 'all the time' to 'rarely' on one or more of the five questions related to medicine taking. 11.4% (95% CI: 8.8-13.9) of patients reported experiencing unmet needs and needing more help with at least one of the aspects of taking medication, in that they ticked 'yes' to the question "do you feel you need more help", on one or more of the five questions related to medicine taking.

Among participants help was needed to some degree with prescriptions and collection of medicines (49.7%), getting medicines out of the box or packet (27.9%), reminding to take medicines (36.4%), swallowing medicines (20.2%) and checking that medicines have been taken (34.2%). Being reminded to take medicines, dealing with prescriptions and collection of medicines and getting medicines out of a pack or bottle were the most commonly reported areas of unmet needs. Almost two thirds of participants (65.3%) reported never missing medicines in the last 30 days. Out of the 34.7% of patients who said they missed taking medicine at any point in the previous 30 days, 23.9% said rarely, 9.3% sometimes, 0.8% often and 0.7% all the time.

Table 2 here

Factors associated with unmet needs

Being on a higher total number of daily medications (OR: 1.2, (1.1-1.3), p<0.001), dependent for ADL (OR: 4.9, (1.9-13.0), p=0.001) and receiving any kind of help (OR: 5.6, (2.7-11.63), p<0.001) in relation to taking medication was associated with experiencing unmet needs. Getting help with swallowing medicines (OR: 6.8, (3.8-12.02), p<0.001), getting medicines out of a box, blister packs or bottles (OR: 6.5,

For peer review only - http://bmjopen.bmj.com/site/about/guidelines.xhtml

(3.6-11.8), p<0.001) showed the strongest associations with experiencing unmet needs. However age, gender, and number of years since stroke showed no significant association with unmet needs (see table 3).

Table 3 here

Factors associated with missing medications

Being older (age ≥70) was associated with a lower probability of missing medication (OR: 0.59 (0.41-0.86) p=0.006). Being on a higher number of daily medicines (polypharmacy) (OR:1.07 (1.02-1.12), p=0.008) and getting any kind of help with medicine taking (OR:2.08 (1.43-3.03) p<0.001) was associated with higher probability of missing medicines. The more unmet needs stroke survivors had with taking medication, the more likely they were to miss their medicines (OR:5.3 (2.9-9.5), p<0.001). Gender, dependence for ADL's and number of years since stroke showed no significant association with missing medicines (see table 4).

Table 4 here

Discussion

Summary of findings

From workshops we identified 5 key issues that patients regarded as important with medication taking after stroke. We converted these into a five item questionnaire that we distributed to people on stroke registers in 18 general practices. We obtained a response rate of 35%. Among respondents, 56% of survivors in the community were receiving help in some aspect of daily medication taking, 11% reported needing more help in at least one domain of medicine taking and 33% missed taking their medicines at some point in the previous 30 days.

For peer review only - http://bmjopen.bmj.com/site/about/guidelines.xhtml

A higher total number of daily medicines, being dependent for ADLs and receiving help with medication were predictors of experiencing at least one unmet need in respect of medication taking. Stroke survivors who were younger, taking a higher number of daily medicines and experiencing a greater number of unmet needs were more likely to miss medications.

This work identified issues from a population that includes patients severely affected by stroke, who are often excluded from research.[17] Results presented here shed light on the effect of stroke related impairments on practical domains and predictors of medicine taking, which are shown to have significant effects on overall adherence.

Strengths and limitations

A strength of this study is that the questionnaire was developed from patients' and caregivers' own views gathered through workshops. Although not recruited through purposive sampling, workshop participants suffered from a range of stroke related impairments, as highlighted by the reported use of Dossette boxes, dependence on others for aspects of medicine taking like prompting medication times, and dependence for ADL's such as collecting prescriptions and taking tablets out of boxes. In the actual postal survey, the inclusion of stroke survivors regardless of level of dependence for ADL's permitted investigating a population who are understudied,[17] yet may have significant unmet needs that can affect their adherence to medications. This investigation highlights caregivers' role in managing medicines in survivors dependent for ADLs.

However, study limitations should also be considered. The response rate across recruited GP practices was low and harder to reach stroke survivors may have been missed. This is a source of bias that might affects our estimates. Through the Barthel score, we did not assess cognition directly, although low cognitive function is associated with poor adherence.[31] We did not collect information on the use of blister packaged medication or devices to aid compliance, which could have influenced medication taking practices. Finally this study examined all medicine taking and did not differentiate between stroke secondary prevention medications and other drug categories.

Comparisons with existing research

For peer review only - http://bmjopen.bmj.com/site/about/guidelines.xhtml

To our knowledge this is the first study that shows that more than half of all stroke survivors get help with some aspect of medicine taking and that those receiving help are more likely to have unmet needs. This provides some insight in to why adherence to medication in stroke survivors may be poor.[32]

Moreover, the greater the number of medicines, the more likely stroke survivors were to miss medications. Addressing pill burden by simplifying drug regimens may be an important focus for future interventions. Indeed the polypill approach to medication taking has been shown to reduce cardiovascular as well as total pill burden in a primary care setting.[33] Simpler dosing regimens, are known to be associated with better medication adherence [34] while fewer medicines has been shown to be an independent predictor of long term medication persistence among stroke survivors.[35 36] A recent trial incorporating a fixed-dose combination polypill approach to taking cardiovascular medicine demonstrated better adherence among patients receiving a single pill.[37]

Receiving help with prescriptions and collecting medicines was identified as the area where most help was received (49.7% of respondents). Stroke survivors who are dependent for activities of daily living may face considerable practical challenges accessing health care resources at the pharmacy and the GP practice. A recent study in the USA found that around 2/3 of caregivers were involved in at least 1 medication management activity of elderly patients and that high involvement in Instrumental Activities of Daily Living (IADLs) was associated with the caregiver providing the patient with assistance in ordering medicines.[38] Filling prescriptions is also known to be an important factor influencing medication adherence.[39 40] Indeed caregivers can play a significant role in ensuring appropriate medication taking. A recent interview study exploring potential barriers and facilitators of medication adherence in stroke identified the central role of the caregiver in medication adherence.[41] Our evaluation of an online stroke forum also confirmed the important role of the caregiver in facilitating medication adherence.[14] Monitoring prescription collections, liaising with the GP and pharmacy, increasing the time between prescriptions or arranging medication deliveries, may help to address prescription needs.

Around 11% of stroke survivors reported unmet medication needs. We found that stroke survivors dependent for ADLs and receiving help with medicines were more likely to report unmet needs, which is in line with a recent study investigating stroke/TIA survivors in Australia, where greater functional ability was associated with fewer unmet needs, including those related to secondary prevention.[42] In previous research on

For peer review only - http://bmjopen.bmj.com/site/about/guidelines.xhtml

unmet needs among stroke survivors, a 44 item survey study by McKevitt and colleagues (2011) reported that 49% of stroke survivors had at least one unmet need [24], while in a study of Australian survivors who completed a 58 item survey, the percentage was 84%.[23] Both these studies however examined unmet needs over a variety of domains including health, work, leisure and everyday living, social support and finances, whereas our study focused on medication needs only.

Getting help to take medicines out of a box, packet or bottle was the area where the greatest proportion of stroke survivors needed help all of the time. We previously found that the use of pill boxes and blister packed medication to be both a facilitator [35] and a barrier [14] to adherence among stroke survivors[15], while interventions using blister packaging and pill boxes have been found to be associated with improved adherence.[43] Although electronic medication devices were considered potentially effective in improving medication taking behaviour among patients with cognitive impairments, success in using such devices was dependent on the patient having a good level of dexterity, while removing the medication from these devices was also found to be challenging. [44 45 46]

The need for further support in this domain, as reported in the current study, suggests that handling medications remains problematic for stroke survivors.

An interesting finding from this survey study is that stroke survivors who missed medicines were younger. This is consistent with other research on adherence in stroke that found that younger age was predictive of poor adherence[47], and has also been described in patients taking medication for cardiovascular disease.[48] The finding in the present study contrasts with the view that older patients are more likely to face difficulties taking medication[49 50] which is frequently attributed to higher number of pre-existing comorbidities resulting in polypharmacy and increased complexity of medication taking regimens. The fact that older patients may be less likely miss medicine might be down to the support they receive from caregivers. Our findings suggests that support needed with medications may be overlooked in younger stroke survivors.[51]

In this study a significant proportion of patients admitted missing medications occasionally. There is evidence that improving adherence by one anti-hypertensive pill/week for a once-a-day regimen reduces the hazard of stroke by 8–9 % and death by 7 %.[52] Each incremental 25% increase in proportion of days covered with statin medications is associated with a 0.10 mmol/L reduction in LDL-C cholesterol.[53 54] Non-adherence to cardiovascular medications is associated with increased risk of morbidity and mortality.[55]

For peer review only - http://bmjopen.bmj.com/site/about/guidelines.xhtml

For peer review only

Implications for clinical practice

A significant proportion of patients, particularly those who take large numbers of tablets, are disabled or receive help to take medication, have unmet needs and miss their tablets, which can increase risk of recurrent cardiovascular events. These particularly vulnerable groups of patients might benefit from focused clinical attention. Through understanding the needs of survivors and caregivers in different aspects of daily medication taking, we can help direct future resources to the areas of greatest need. For example, further exploration of medication packaging is warranted to understand the difficulties stroke survivors face handling medicines. Polypharmacy remains a difficulty for older patients. Therefore, exploring the use of combination pills and further efforts to reduce the burden of multiple medications among stroke survivors is warranted.

The questionnaire we have developed could be used to understand the challenges around medication faced by other patient groups Unmet medication needs among UK stroke survivors have not been previously explored in the context of activities both survivors and caregivers consider important for taking medicines. Through understanding the extent of unmet needs as well as the areas in which these are greatest, strategies can be developed which address poor medication taking practices and therefore improve medication adherence.

Future research

Novel interventions focussing on the practicalities of taking medicines and aimed at improving stroke survivors' adherence to treatment are needed. The findings reported here may inform the development of such interventions. Advances in technology have the potential to facilitate delivery of such interventions, e.g. electronic devices prompting medication taking times.[56 57] Efforts to improve medication taking among survivors of stroke using technology are already underway and have shown promise.[58]

For peer review only - http://bmjopen.bmj.com/site/about/guidelines.xhtml

Acknowledgements

The authors wish to thank all the stroke survivors and caregivers who participated in this study.

Competing interests

None declared

Data sharing statement

No additional data are available

Author/s contribution

ADS is the Chief Investigator, contributed to the study design, data analysis and commented on the manuscript. JJ contributed to the study design, data collection, data analysis and prepared the manuscript for submission. JM is a co-investigator on the study, wrote and commented on the manuscript. SS is a co-investigator on the study, wrote and commented on the manuscript. LA contributed to the data analysis and commented on the manuscript. GDiT contributed to the data analysis and commented on the manuscript. All authors agreed on the final draft of the submitted manuscript.

References

1. Marek KD AL. Medication Management of the Community-Dwelling Older Adult. In: RG H, ed. An Evidence-Based Handbook for Nurses: Agency for Healthcare Research and Quality (US). Rockville, MD2008.

2. Lewis A. Non-compliance: A $100 billion problem. . *The Remington Report* 1997;5:14-5.

3. office NA. Progress in improving stroke care. In: Office NA, ed. London: Department of Health, 2010.

4. Young J, Forster A. Review of stroke rehabilitation. *BMJ* 2007;334(7584):86-90. doi: 10.1136/bmj.39059.456794.68

5. Miller EL, Murray L, Richards L, et al. Comprehensive overview of nursing and interdisciplinary rehabilitation care of the stroke patient: a scientific statement from the American Heart Association. *Stroke* 2010 (Oct);41(10):2402-48. doi: 10.1161/STR.0b013e3181e7512b

For peer review only - http://bmjopen.bmj.com/site/about/guidelines.xhtml

6. Feigin VL, Barker-Collo S, Parag V, et al. Auckland Stroke Outcomes Study: Part 1: Gender, stroke types, ethnicity, and functional outcomes 5 years poststroke. *Neurology* 2010;75(18):1597-607. doi: 10.1212/WNL.0b013e3181fb44b3

7. Park JH, Park JH, Lee SY, et al. Disparities in Antihypertensive Medication Adherence in Persons With Disabilities and Without Disabilities: Results of a Korean Population-Based Study. *Archives of Physical Medicine and Rehabilitation* 2008;89(8):1460-67. doi: http://dx.doi.org/10.1016/j.apmr.2007.12.045

8. Stilley CS, Bender CM, Dunbar-Jacob J, et al. The Impact of Cognitive Function on Medication Management: Three Studies. *Health psychology : official journal of the Division of Health Psychology, American Psychological Association* 2010;29(1):50-55. doi: 10.1037/a0016940

9. Smith D, Lovell J, Weller C, et al. A systematic review of medication non-adherence in persons with dementia or cognitive impairment. *PLoS ONE* 2017;12(2):e0170651. doi: 10.1371/journal.pone.0170651

10. Kronish IM, Ye S. Adherence to Cardiovascular Medications: Lessons Learned and Future Directions. *Progress in cardiovascular diseases* 2013;55(6):590-600. doi: 10.1016/j.pcad.2013.02.001

11. Gordon K, Smith F, Dhillon S. Effective chronic disease management: Patients' perspectives on medication-related problems. *Patient Education and Counseling* 2007;65(3):407-15. doi: http://dx.doi.org/10.1016/j.pec.2006.09.012

12. Xu J, Zhao X, Wang Y, et al. Impact of a Better Persistence with Antihypertensive Agents on Ischemic Stroke Outcomes for Secondary Prevention. *PLoS ONE* 2013;8(6):e65233. doi: 10.1371/journal.pone.0065233

13. Colivicchi F, Bassi A, Santini M, et al. Discontinuation of Statin Therapy and Clinical Outcome After Ischemic Stroke. *Stroke* 2007;38(10):2652-57. doi: 10.1161/strokeaha.107.487017

14. Jamison J, Sutton S, Mant J, et al. Barriers and facilitators to adherence to secondary stroke prevention medications after stroke: Analysis of survivors and caregivers views from an online stroke forum. *BMJ Open* 2017;7 (e016814)

For peer review only

15. Jamison J, Graffy J, Mullis R, et al. Barriers to medication adherence for the secondary prevention of stroke: a qualitative interview study in primary care. *The British Journal of General Practice* 2016;66(649):e568-e76. doi: 10.3399/bjgp16X685609

16. Souter C, Kinnear A, Kinnear M, et al. Optimisation of secondary prevention of stroke: a qualitative study of stroke patients' beliefs, concerns and difficulties with their medicines. *International Journal of Pharmacy Practice* 2014;22(6):424-32. doi: 10.1111/ijpp.12104

17. De Simoni A, Mant J, Sutton S. Adherence to medication in stroke survivors dependent on caregivers. *British Journal of General Practice* 2015;65(640):e789.

18. De Simoni A, Hardeman W, Mant J, et al. Trials to Improve Blood Pressure Through Adherence to Antihypertensives in Stroke/TIA: Systematic Review and Meta-Analysis. *Journal of the American Heart Association* 2013 (Aug 20);2(4):e000251. doi: 10.1161/jaha.113.000251

19. Munger MA, Van Tassell BW, LaFleur J. Medication Nonadherence: An Unrecognized Cardiovascular Risk Factor. *Medscape General Medicine* 2007;9(3):58-58.

20. Brown MT, Bussell JK. Medication Adherence: WHO Cares? *Mayo Clinic proceedings* 2011;86(4):304-14. doi: 10.4065/mcp.2010.0575

21. Wolff JL, Spillman B. Older Adults Receiving Assistance With Physician Visits and Prescribed Medications and Their Family Caregivers: Prevalence, Characteristics, and Hours of Care. *The Journals of Gerontology: Series B* 2014;69(Suppl_1):S65-S72. doi: 10.1093/geronb/gbu119

22. Ownby RL, Hertzog C, Crocco E, et al. Factors related to medication adherence in memory disorder clinic patients. *Aging & mental health* 2006;10(4):378-85. doi: 10.1080/13607860500410011

23. Andrew NE, Kilkenny M, Naylor R, et al. Understanding Long-Term Unmet Needs in Australian Survivors of Stroke. *International Journal of Stroke* 2014;9(SA100):106-12. doi: doi:10.1111/ijs.12325

24. McKevitt C, Fudge N, Redfern J, et al. Self-Reported Long-Term Needs After Stroke. *Stroke* 2011;42(5):1398-403. doi: 10.1161/strokeaha.110.598839

25. De Simoni A KI, Sutton S, Farmer A, Blenkinsopp A, Kinmonth AL, Mant J. Developing a pharmacist intervention to improve adherence to hypertensive medications in TIA/stroke patients. SAPC Conference. Norwich, 2010.

For peer review only - http://bmjopen.bmj.com/site/about/guidelines.xhtml

26. Braun V, Clarke V. Using thematic analysis in psychology. *Qualitative Research in Psychology* 2006;3(2):77-101. doi: 10.1191/1478088706qp063oa

27. Gompertz P, Pound P, Ebrahim S. A postal version of the Barthel Index. *Clinical Rehabilitation* 1994;8(3):233-39. doi: doi:10.1177/026921559400800308

28. Quinn TJ, Langhorne P, Stott DJ. Barthel Index for Stroke Trials. *Development, Properties, and Application* 2011;42(4):1146-51. doi: 10.1161/strokeaha.110.598540

29. Document Capture Company [Available from: http://www.documentcapture.co.uk/data-collection-methods/

30. Tom Smith MN, Stefan Noble, Gemma Wright, David McLennan and Emma Plunkett. The English Indices of Deprivation 2015 Research report, 2015.

31. Hayes TL, Larimer N, Adami A, et al. Medication Adherence in Healthy Elders. *Journal of Aging and Health* 2009;21(4):567-80. doi: 10.1177/0898264309332836

32. Chowdhury R, Khan H, Heydon E, et al. Adherence to cardiovascular therapy: a meta-analysis of prevalence and clinical consequences. *European Heart Journal* 2013;34:2940-8. doi: 10.1093/eurheartj/eht295

33. Truelove M, Patel A, Bompoint S, et al. The Effect of a Cardiovascular Polypill Strategy on Pill Burden. *Cardiovascular Therapeutics* 2015;33(6):347-52. doi: 10.1111/1755-5922.12151

34. Claxton AJ, Cramer, J., Pierce, C. A Systematic Review of the Associations Between Dose Regimens and Medication Compliance *Clinical therapeutics* 2001;23(8):1296-310.

35. Bushnell CD, Olson DM, Zhao X, et al. Secondary preventive medication persistence and adherence 1 year after stroke. *Neurology* 2011;77(12):1182-90. doi: 10.1212/WNL.0b013e31822f0423 [published Online First: 2011/09/09]

36. Chapman RH, Benner JS, Petrilla AA, et al. Predictors of adherence with antihypertensive and lipid-lowering therapy. *Archives of Internal Medicine* 2005;165(10):1147-52. doi: 10.1001/archinte.165.10.1147

37. Thom S, Poulter N, Field J, et al. Effects of a fixed-dose combination strategy on adherence and risk factors in patients with or at high risk of CVD: the UMPIRE randomized clinical trial. *JAMA : the journal of the American Medical Association* 2013;310(9):918-29. doi: 10.1001/jama.2013.277064

For peer review only - http://bmjopen.bmj.com/site/about/guidelines.xhtml

38. Noureldin M, Plake KS. Correlates of caregivers' involvement in the management of older adults' medications. *Research in Social and Administrative Pharmacy* 2017;13(4):840-48. doi: https://doi.org/10.1016/j.sapharm.2016.09.009

39. Andersson K, Melander A, Svensson C, et al. Repeat prescriptions: refill adherence in relation to patient and prescriber characteristics, reimbursement level and type of medication. *European Journal of Public Health* 2005;15(6):621-26. doi: 10.1093/eurpub/cki053

40. Vlasnik JJ, Aliotta SL, DeLor B. Medication adherence: Factors influencing compliance with prescribed medication plans. *The Case Manager* 2005;16(2):47-51. doi: http://dx.doi.org/10.1016/j.casemgr.2005.01.009

41. Bauler S, Jacquin-Courtois S, Haesebaert J, et al. Barriers and Facilitators for Medication Adherence in Stroke Patients: A Qualitative Study Conducted in French Neurological Rehabilitation Units. *European Neurology* 2014;72(5-6):262-70.

42. Olaiya MT, Cadilhac DA, Kim J, et al. Long-term unmet needs and associated factors in stroke or TIA survivors: An observational study. *Neurology* 2017 (Jul 4);89(1):68-75. doi: 10.1212/wnl.0000000000004063

43. Conn VS, Ruppar TM, Chan KC, et al. Packaging interventions to increase medication adherence: systematic review and meta-analysis. *Current Medical Research and Opinion* 2015;31(1):145-60. doi: 10.1185/03007995.2014.978939

44. Hall J, Bond C, Kinnear M, et al. Views of patients and professionals about electronic multicompartment medication devices: a qualitative study. *BMJ Open* 2016;6:e012915. doi: 10.1136/bmjopen-2016-012915

45. Beckman AGK, Parker MG, Thorslund M. Can elderly people take their medicine? *Patient Education and Counseling* 2005;59(2):186-91. doi: http://dx.doi.org/10.1016/j.pec.2004.11.005

46. Notenboom K, Beers E, van Riet-Nales DA, et al. Practical Problems with Medication Use that Older People Experience: A Qualitative Study. *Journal of the American Geriatrics Society* 2014;62(12):2339-44. doi: 10.1111/jgs.13126

For peer review only - http://bmjopen.bmj.com/site/about/guidelines.xhtml

47. O'Carroll R, Whittaker J, Hamilton B, et al. Predictors of adherence to secondary preventive medication in stroke patients. *Ann Behav Med* 2011;41(3):383-90. doi: 10.1007/s12160-010-9257-6 [published Online First: 2011/01/05]

48. Crowley MJ, Zullig LL, Shah BR, et al. Medication Non-Adherence After Myocardial Infarction: An Exploration of Modifying Factors. *Journal of General Internal Medicine* 2015;30(1):83-90. doi: 10.1007/s11606-014-3072-x

49. Hughes CM. Medication Non-Adherence in the Elderly. *Drugs & Aging* 2004;21(12):793-811. doi: 10.2165/00002512-200421120-00004

50. De Schryver EL, van Gijn J, Kappelle LJ, et al. Non-adherence to aspirin or oral anticoagulants in secondary prevention after ischaemic stroke. *J Neurol* 2005;252(11):1316-21. doi: 10.1007/s00415-005-0858-0 [published Online First: 2005/05/04]

51. Kersten P, Low JTS, Ashburn A, et al. The unmet needs of young people who have had a stroke: results of a national UK survey. *Disability and Rehabilitation* 2002;24(16):860-66. doi: 10.1080/09638280210142167

52. Bailey JE, Wan JY, Tang J, et al. Antihypertensive medication adherence, ambulatory visits, and risk of stroke and death. *J Gen Intern Med* 2010;25(6):495-503. doi: 10.1007/s11606-009-1240-1 [published Online First: 2010/02/19]

53. Amarenco P, Labreuche J. Lipid management in the prevention of stroke: review and updated meta-analysis of statins for stroke prevention. *The Lancet Neurology* 2009;8(5):453-63. doi: 10.1016/s1474-4422(09)70058-4 [published Online First: 2009/04/21]

54. Ho PM, Rumsfeld JS, Masoudi FA, et al. Effect of medication nonadherence on hospitalization and mortality among patients with diabetes mellitus. *Arch Intern Med* 2006;166(17):1836-41. doi: 10.1001/archinte.166.17.1836 [published Online First: 2006/09/27]

55. Rasmussen JN, Chong A, Alter DA. Relationship between adherence to evidence-based pharmacotherapy and long-term mortality after acute myocardial infarction. *Jama* 2007;297(2):177-86. doi: 10.1001/jama.297.2.177 [published Online First: 2007/01/11]

For peer review only - http://bmjopen.bmj.com/site/about/guidelines.xhtml

56. Tao D, Xie L, Wang T, et al. A meta-analysis of the use of electronic reminders for patient adherence to medication in chronic disease care. *Journal of Telemedicine and Telecare* 2015;21(1):3-13. doi: doi:10.1177/1357633X14541041

57. Thakkar J, Kurup R, Laba T, et al. Mobile telephone text messaging for medication adherence in chronic disease: A meta-analysis. *JAMA Internal Medicine* 2016;176(3):340-49. doi: 10.1001/jamainternmed.2015.7667

58. Kamal AK, Shaikh Q, Pasha O, et al. A randomized controlled behavioral intervention trial to improve medication adherence in adult stroke patients with prescription tailored Short Messaging Service (SMS)-SMS4Stroke study. *BMC Neurology* 2015;15(1):212. doi: 10.1186/s12883-015-0471-5

For peer review only

For peer review only - http://bmjopen.bmj.com/site/about/guidelines.xhtml

		Age (years)	Female	Male	Time since stroke (Years)	N of daily medicines	Independent for ADLs (BI=20)
All patients	N	588	210	346	535	557	139
	%		37.77	62.23			28.31
	Mean	72.69			7.65	6.38	
	SD	11.57			7.58	4.00	
Patients who receive help of any kind	N	331	112	197	295	312	53
	%		36.25	63.25			18.93
	Mean	73.57			7.97	7.33	
	SD	12.23			8.50	4.45	

Table 1. Characteristics of study participants *(mean scores reported unless otherwise stated).*N represent the number of participants who completed the survey in respect to the different variables.

For peer review only - http://bmjopen.bmj.com/site/about/guidelines.xhtml

	N	All the Time N (%)	Often N (%)	Sometimes N (%)	Rarely N (%)	Never N (%)	Yes N (%)	No N (%)
Question 1<br>Is somebody helping with prescriptions and collection of your medicines?	583	186 *(31.9)*	19 *(3.3)*	40 *(6.9)*	45 *(7.7)*	293 *(50.2)*		
Question 1a<br>Do you feel you need more help with prescriptions and collection of your medicines?	551						33 *(6.0)*	518 *(94.0)*
Question 2<br>Is somebody helping you getting the medicines out of the box, bottle or blister pack?	578	85 *(14.7)*	15 *(2.6)*	31 *(5.4)*	30 *(5.2)*	417 *(72.1)*		
Question 2a<br>Do you feel you need more help with getting the medicines out of the box, bottle or blister pack?	553						33 *(6.0)*	520 *(94.0)*
Question 3<br>Is somebody helping with reminding you when is the time to take your medicine?	577	78 *(13.6)*	22 *(3.8)*	59 *(10.2)*	51 *(8.8)*	367 *(63.6)*		
Question 3a<br>Do you feel you need more help with reminding when is the time to take your medicine?							35 *(6.2)*	529 *(93.8)*
Question4<br>Is somebody helping you with swallowing your medicine?	579	56 *(9.7)*	11 *(1.9)*	29 *(5.0)*	21 *(3.6)*	462 *(79.8)*		
Question 4a<br>Do you feel you need more help with swallowing your medicine?							9 *(1.6)*	551 *(98.4)*
Question 5<br>I somebody helping you with checking that you have taken your medicines	576	76 *(13.2)*	23 *(4.0)*	58 *(10.0)*	40 *(6.9)*	379 *(65.9)*		
Question 5 a<br>Do you feel you need more help with checking that you have taken your medicine							20 *(3.6)*	538 *(96.4)*
Thinking of the last 30 days, how often did you miss taking your regular medicines?	594	4 *(0.7)*	5 *(0.8)*	55 *(9.3)*	142 *(23.9)*	388 *(65.3)*		

Table 2. Responses to the survey questions.

For peer review only

	Univariable analysis	Multivariable analysis
**Variable**	**Odds ratio (95% CI) p value**	**Odds ratio (95% CI) p value**
Age ≥70	0.63 (0.37-1.06) p=0.084	0.69 (0.40-1.19) p=0.180
Gender (female)	0.65 (0.36-1.15) p=0.137	0.65 (0.36-1.17) p=0.147
Number of different medicines	1.20 (1.12-1.28) p<0.001	1.22 (1.14-1.31) p<0.001
Dependence for ADLs	4.10 (1.59-10.56) p=0.003	4.91 (1.86-12.97) p=0.001
Years since stroke	1.03 (1.00-1.06) p=0.078	1.02 (0.99-1.05) p=0.160
Getting help with prescriptions and collection of medication	4.67 (2.48-8.79) p<0.001	4.55 (2.38-8.67) p<0.001
Getting help with taking medicines out of the box, bottle or blister pack	6.70 (3.81-11.77) p<0.001	6.53 (3.63-11.75) p<0.001
Getting help with reminding you when is the time to take your medicine?	4.70 (2.68-8.21) p<0.001	4.25 (2.39-7.56) p<0.001
Getting help to swallow the medication	6.72 (3.89-11.59) p<0.001	6.75 (3.79-12.02) p<0.001
Getting help by checking that you have taken your medicines	4.89 (2.81-8.52) p<0.001	5.58 (3.08-10.09) p<0.001
Getting any kind of help	5.86 (2.84-12.08) p<0.001	5.58 (2.68-11.63) p<0.001

Table 3. Variables associated with unmet needs.

For peer review only

	**Univariable analysis**	**Multivariable analysis**
**Variable**	**Odds Ratio (95% CI) p value**	**Odds Ratio (95% CI) p value**
Age ≥70	0.59 (0.42-0.84) p=0.003	0.59 (0.41-0.86) p=0.006
Gender (female)	0.86 (0.59-1.23) p=0.401	0.88 (0.61-1.28) p=0.498
Number of different medicines	1.04 (1.00-1.09) p=0.040	1.07 (1.02-1.12) p=0.008
Dependence for ADLs (BI<20)	1.21 (0.80-1.84) p=0.362	1.30 (0.83-2.04) p=0.248
Years since stroke	1.00 (0.98-1.02) p=0.950	1.00 (0.98-1.02) p=0.971
Getting help with prescriptions and collection of medication	2.04 (1.45-2.90) p<0.001	2.29 (1.58-3.32) p<0.001
Getting help to have the medicines out of the box, bottle or blister pack	1.39 (0.95-2.02) p=0.089	1.49 (1.00-2.21) p=0.051
Getting help with reminding you when is the time to take your medicine?	2.48 (1.74-3.55) p<0.001	2.67 (1.83-3.90) p<0.001
Getting help to swallow the medication	1.53 (1.01-2.32) p=0.045	1.68 (1.08-2.61) p=0.022
Getting help by checking that you have taken your medicines	2.37 (1.66-3.39) p<0.001	2.50 (1.70-3.66) p<0.001
Getting any kind of help	2.06 (1.44-2.93) p<0.001	2.08 (1.43-3.03) p<0.001
Unmet needs (participant reported more help needed)	5.27 (3.02-9.22) p<0.000	5.09 (2.84-9.11) p<0.001

Table 4. Variables associated with missing medicines.

For peer review only

For peer review only - http://bmjopen.bmj.com/site/about/guidelines.xhtml

University of London

UNIVERSITY OF CAMBRIDGE

QUESTIONNAIRE – PATIENT

Many people with stroke need help with taking their medicines. We would like to ask you few questions about the help you might need with taking your medicines.

How many different type of medicines do you take in one day? ☐
Write the number or an approximation. Please count all medications including eye drops, injections etc.

How old are you? ☐ How long ago was your stroke? ☐ years

What is your sex? M ☐ F ☐

For each question below, please tick the box that best describes how you have taken your medicines <u>in the last month</u>:

1. **Is somebody helping with prescriptions and collection of your medicines?**

   all the time ☐
   often ☐
   sometimes ☐
   rarely ☐
   never ☐

   Do you feel you need more help with prescriptions and collection of your medicines?

   Yes ☐ **No** ☐

2. **Is somebody helping you getting the medicines out of the box, bottle or blister pack?**

   all the time ☐
   often ☐
   sometimes ☐
   rarely ☐
   never ☐

   Do you feel you need more help with getting the medicines out of the box, bottle or blister pack?

   Yes ☐ **No** ☐

For peer review only

3. Is somebody helping with reminding you when is the time to take your medicine?

all the time ☐
often ☐
sometimes ☐
rarely ☐
never ☐

Do you feel you need more help with reminding when is the time to take your medicine?

Yes ☐ **No** ☐

4. Is somebody helping you with swallowing your medicine? For example by giving you a drink

all the time ☐
often ☐
sometimes ☐
rarely ☐
never ☐

Do you feel you need more help with swallowing your medicine?

Yes ☐ **No** ☐

5. Is somebody helping you with checking that you have taken your medicines?

all the time ☐
often ☐
sometimes ☐
rarely ☐
never ☐

Do you feel you need more help with checking that you have taken your medicine?

Yes ☐ **No** ☐

Missing medicines

Thinking of the last 30 days, how often did you miss taking your regular medicines?

Remember - tick one box only

all the time ☐
often ☐
sometimes ☐
rarely ☐
never ☐

For peer review only

Barthel Questionnaire

These are some questions about your ability to look after yourself.

They may not seem to apply to you.

Please answer them all.

Tick one box in each section.

Bathing

In the bath or shower do you:

Remember - tick one box only

- manage on your own? ☐
- need help getting in and out? ☐
- need other help? ☐
- never have a bath or shower? ☐
- need to be washed in bed? ☐

Stairs

Do you climb stairs at home:

Remember - tick one box only

- without any help? ☐
- with someone carrying your frame? ☐
- with someone encouraging you? ☐
- with physical help? ☐
- not at all? ☐
- don't have stairs? ☐

Dressing

Do you get dressed:

Remember - tick one box only

- without any help? ☐
- just with help with buttons? ☐
- with someone helping you most of the time? ☐

Mobility

Do you walk indoors:

Remember - tick one box only

- without any help apart from a frame? ☐
- with one person watching over you? ☐
- with one person helping you? ☐
- with more than one person helping? ☐
- not at all? ☐
- Or do you use a wheelchair independently? (e.g. round corners) ☐

Transfer

Do you move from bed to chair:

Remember - tick one box only

- on your own? ☐
- with a little help from one person? ☐
- with a lot of help from one or more people? ☐
- not at all? ☐

For peer review only

Feeding

Do you eat food:

Remember - tick one box only

- without any help? ☐
- with help cutting food or spreading butter? ☐
- with more help? ☐

Toilet use

Do you use a toilet or commode:

Remember - tick one box only

- without any help? ☐
- with some help but can do something? ☐
- with quite a lot of help? ☐

Grooming

Do you brush your hair and teeth
Wash your face and shave:

Remember - tick one box only

- without help? ☐
- with help? ☐

Bladder

Are you incontinent of urine?

Remember - tick one box only

- never ☐
- less than once a week ☐
- less than once a day ☐
- more often ☐
- Or do you have a catheter managed for you ☐

Bowels

Do you soil yourself?

Remember - tick one box only

- never ☐
- Occasional accident ☐
- all the time ☐
- or do you need someone to give you an enema? ☐

For peer review only

□□.□□□

Queen Mary University of London — UNIVERSITY OF CAMBRIDGE

Help with taking tablets after stroke

FAMILY MEMBER/FRIEND OR PRIVATE CARER COPY

For peer review only

Many people with stroke need help with taking their medicines. We would like to ask you few questions about the help you might be offering to your family member/friend/ patient with stroke with taking medicines.

Relation with your family member/friend with stroke

Are you:

Remember - tick one box only

- partner □
- son or daughter □
- friend □
- carer from an agency □
- other □
- if other, please specify

How many different types of medicines does your family member/friend/patient with stroke take in one day? □
Write the number or an approximation. Please count all medications including eye drops, injections etc.

How old is your family member/friend/patient with stroke? (years) □

How many years ago was your family member/friend/patient's stroke? □

What is your family member/friend/patient with stroke sex? M □ F □

For each question below, please tick the box that best describes the help needed by your family member/friend/patient with stroke with taking medicines in the last month.

1. **Is somebody helping your family member/friend/patient with stroke with prescriptions and collection of his/her medicines?**
   - all the time □
   - often □
   - sometimes □
   - rarely □
   - never □

   Do you feel your family member/friend/patient with stroke needs more help with prescriptions and collection of his/her medicines?

   Yes □ **No** □

Version 1.0 010316

☐ ☐ . ☐ ☐ ☐

2. Is somebody helping your family member/friend/patient with stroke getting the medicines out of the box, bottle or blister pack?

all the time ☐
often ☐
sometimes ☐
rarely ☐
never ☐

Do you feel your family member/friend/patient with stroke needs more help with getting the medicines out of the box, bottle, or blister pack?

Yes ☐ **No** ☐

3. Is somebody helping your family member/friend/patient with stroke with reminding when is time to take his/her medicine?

all the time ☐
often ☐
sometimes ☐
rarely ☐
never ☐

Do you feel your family member/friend/patient with stroke needs more help with reminding when is time to take his/her medicine?

Yes ☐ **No** ☐

4. Is somebody helping your family member/friend/patient with stroke with swallowing his/her medicine? For example by giving a drink.

all the time ☐
often ☐
sometimes ☐
rarely ☐
never ☐

Do you feel you your family member/friend/patient with stroke need more help with swallowing his/her medicine?

Yes ☐ **No** ☐

5. Is somebody helping your family member/friend/patient with stroke with checking that he/she has taken his/her medicines?

all the time ☐
often ☐
sometimes ☐
rarely ☐
never ☐

Do you feel your family member/friend/patient with stroke needs more help with checking that he/she has taken his/her medicine?

Yes ☐ **No** ☐

Version 1.0 010316

For peer review only - http://bmjopen.bmj.com/site/about/guidelines.xhtml

□□.□□□

Missing medicines

Thinking of the last 30 days, how often did your family member/friend/patient with stroke miss taking his/her regular medicines?

Remember - tick one box only

- all the time ☐
- often ☐
- sometimes ☐
- rarely ☐
- never ☐

Barthel Questionnaire

These are some questions about the ability of your family member/friend/patient with stroke to look after him/herself.

Please answer them all.

Please fill this questionnaire even if you are not regularly caring for your family member/friend/patient with stroke, trying to answer questions in the way you think most accurately describes the disability of your family member/friend/patient with stroke.

Tick one box in each section.

Bathing

In the bath or shower do you:

Remember - tick one box only

- manage on your own? ☐
- need help getting in and out? ☐
- need other help? ☐
- never have a bath or shower? ☐
- need to be washed in bed? ☐

Stairs

Do you climb stairs at home:

Remember - tick one box only

- without any help? ☐
- with someone carrying your frame? ☐
- with someone encouraging you? ☐
- with physical help? ☐
- not at all? ☐
- don't have stairs? ☐

Dressing

Do you get dressed:

Remember - tick one box only

- without any help? ☐
- just with help with buttons? ☐
- with someone helping you most of the time? ☐

Mobility

Do you walk indoors:

Remember - tick one box only

- without any help apart from a frame? ☐
- with one person watching over you? ☐
- with one person helping you? ☐

Version 1.0 010316

For peer review only

	with more than one person helping? ☐
	not at all? ☐
	Or do you use a wheelchair independently? ☐ (e.g. round corners)

Transfer

Do you move from bed to chair:	on your own? ☐
	with a little help from one person? ☐
Remember - tick one box only	with a lot of help from one or more people? ☐
	not at all? ☐

Feeding

Do you eat food:	without any help? ☐
	with help cutting food or spreading butter? ☐
Remember - tick one box only	with more help? ☐

Toilet use

Do you use a toilet or commode:	without any help? ☐
	with some help but can do something? ☐
Remember - tick one box only	with quite a lot of help? ☐

Grooming

Do you brush your hair and teeth	without help? ☐
Wash your face and shave:	with help? ☐
Remember - tick one box only	

Bladder

Are you incontinent of urine?	never ☐
	less than once a week ☐
Remember - tick one box only	less than once a day ☐
	more often ☐
	Or do you have a catheter managed for you ☐

Bowels

Do you soil yourself?	never ☐
	Occasional accident ☐
Remember - tick one box only	all the time ☐
	or do you need someone to give you an enema? ☐

Version 1.0 010316

For peer review only

STROBE 2007 (v4) Statement—Checklist of items that should be included in reports of *cross-sectional studies*

Section/Topic	Item #	Recommendation	Reported on page #
**Title and abstract**	1	(*a*) Indicate the study's design with a commonly used term in the title or the abstract	2
		(*b*) Provide in the abstract an informative and balanced summary of what was done and what was found	2
**Introduction**			
Background/rationale	2	Explain the scientific background and rationale for the investigation being reported	4-5
Objectives	3	State specific objectives, including any prespecified hypotheses	5
**Methods**			
Study design	4	Present key elements of study design early in the paper	6
Setting	5	Describe the setting, locations, and relevant dates, including periods of recruitment, exposure, follow-up, and data collection	6
Participants	6	(*a*) Give the eligibility criteria, and the sources and methods of selection of participants	7-8
Variables	7	Clearly define all outcomes, exposures, predictors, potential confounders, and effect modifiers. Give diagnostic criteria, if applicable	8
Data sources/ measurement	8*	For each variable of interest, give sources of data and details of methods of assessment (measurement). Describe comparability of assessment methods if there is more than one group	6-7
Bias	9	Describe any efforts to address potential sources of bias	-
Study size	10	Explain how the study size was arrived at	10
Quantitative variables	11	Explain how quantitative variables were handled in the analyses. If applicable, describe which groupings were chosen and why	8-9
Statistical methods	12	(*a*) Describe all statistical methods, including those used to control for confounding	8-9
		(*b*) Describe any methods used to examine subgroups and interactions	8-9
		(*c*) Explain how missing data were addressed	-
		(*d*) If applicable, describe analytical methods taking account of sampling strategy	-
		(*e*) Describe any sensitivity analyses	-

For peer review only

**Results**			
Participants	13*	(a) Report numbers of individuals at each stage of study—eg numbers potentially eligible, examined for eligibility, confirmed eligible, included in the study, completing follow-up, and analysed	10
		(b) Give reasons for non-participation at each stage	-
		(c) Consider use of a flow diagram	-
Descriptive data	14*	(a) Give characteristics of study participants (eg demographic, clinical, social) and information on exposures and potential confounders	10-11
		(b) Indicate number of participants with missing data for each variable of interest	-
Outcome data	15*	Report numbers of outcome events or summary measures	
Main results	16	(*a*) Give unadjusted estimates and, if applicable, confounder-adjusted estimates and their precision (eg, 95% confidence interval). Make clear which confounders were adjusted for and why they were included	11-12
		(*b*) Report category boundaries when continuous variables were categorized	-
		(*c*) If relevant, consider translating estimates of relative risk into absolute risk for a meaningful time period	-
Other analyses	17	Report other analyses done—eg analyses of subgroups and interactions, and sensitivity analyses	-
**Discussion**			
Key results	18	Summarise key results with reference to study objectives	13
Limitations	19	Discuss limitations of the study, taking into account sources of potential bias or imprecision. Discuss both direction and magnitude of any potential bias	13-14
Interpretation	20	Give a cautious overall interpretation of results considering objectives, limitations, multiplicity of analyses, results from similar studies, and other relevant evidence	14-16
Generalisability	21	Discuss the generalisability (external validity) of the study results	17
**Other information**			
Funding	22	Give the source of funding and the role of the funders for the present study and, if applicable, for the original study on which the present article is based	3

*Give information separately for cases and controls in case-control studies and, if applicable, for exposed and unexposed groups in cohort and cross-sectional studies.

Note: An Explanation and Elaboration article discusses each checklist item and gives methodological background and published examples of transparent reporting. The STROBE checklist is best used in conjunction with this article (freely available on the Web sites of PLoS Medicine at http://www.plosmedicine.org/, Annals of Internal Medicine at http://www.annals.org/, and Epidemiology at http://www.epidem.com/). Information on the STROBE Initiative is available at www.strobe-statement.org.

For peer review only

BMJ Open

Evaluating practical support stroke survivors get with medicines and unmet needs in primary care: A survey

Journal:	*BMJ Open*
Manuscript ID	bmjopen-2017-019874.R1
Article Type:	Research
Date Submitted by the Author:	08-Dec-2017
Complete List of Authors:	JAMISON, JAMES; UNIVERSITY OF CAMBRIDGE, PUBLIC HEALTH & PRIMARY CARE<br>Ayerbe, Luis; Queen Mary University of London, Centre for Primary Care and Public Health<br>Di Tanna, Gian Luca; Queen Mary University of London<br>Sutton, Stephen; University of Cambridge,<br>Mant, Jonathan; University of Cambridge, General Practice and Primary Care Research Unit<br>De Simoni, Anna; Queen Mary University of London, Centre for Primary Care and Public Health
**Primary Subject Heading**:	Cardiovascular medicine
Secondary Subject Heading:	Cardiovascular medicine, Public health, General practice / Family practice
Keywords:	Medication adherence, Caregivers, Barthel, STROKE MEDICINE

only

SCHOLARONE™
Manuscripts

Evaluating practical support stroke survivors get with medicines and unmet needs in primary care: A survey

*James Jamison[1], MSc

Luis Ayerbe[2], PhD

Gian Luca Di Tanna[2] PhD

Stephen Sutton[1], PhD

Jonathan Mant[1], MD

Anna De Simoni[2], PhD

[1]Primary Care Unit, Department of Public Health & Primary Care, Forvie Site, University of Cambridge School of Clinical Medicine, Box 113 Cambridge Biomedical Campus, Cambridge, CB2 0SR.

[2]Centre for Primary Care and Public Health, Barts and The London School of Medicine and Dentistry, Yvonne Carter Building, London E1 2AB.

*corresponding author: James Jamison; Tel: +44 (0)1223 768272. Fax: +44 (0)1223 763492. Email:

jj285@medschl.cam.ac.uk

For peer review only - http://bmjopen.bmj.com/site/about/guidelines.xhtml

Abstract

Objectives

To design a questionnaire and use it to explore unmet needs with practical aspects of medicines taking after stroke, predictors of medicine taking and to estimate the proportion of survivors who get support with daily medication taking.

Design

Four workshops with stroke survivors and caregivers to design the questionnaire.

A cross-sectional postal questionnaire in primary care.

Setting

18 GP practices in the East of England and London. Questionnaires posted between September 2016 and February 2017.

Participants

1687 stroke survivors living in the community outside institutional long term care.

Primary Outcome measures

The proportion of community stroke survivors receiving support from caregivers for practical aspects of medicine taking; the proportion with unmet needs in this respect; the predictors of experiencing unmet needs and missing taking medications.

Results

A 5-item questionnaire was developed to cover the different aspects of medicine taking. 596/1687 (35%) questionnaires were returned. 56% reported getting help in at least one aspect of taking medication and 11% needing more help. 33% reported missing taking their medicines. Unmet needs were associated with receiving help with medications (OR: 5.6, p<0.001), being on a higher number of medications (OR: 1.2, p<0.001) and being dependent for activities of daily living (ADLs) (OR: 4.9, p=0.001). Missing medication was associated with having unmet needs (OR: 5.1, p<0.001), receiving help with medications (OR: 2.1, p<0.001), being on a higher number of medicines (OR: 1.1, p=0.008) and being older than 70 years (OR: 0.6, p=0.006).

For peer review only - http://bmjopen.bmj.com/site/about/guidelines.xhtml

Conclusions

More than half of patients who replied needed help with taking medication, and 1 in 10 had unmet needs in this regard. Stroke survivors dependent on others have more unmet needs, more likely to miss medicines and might benefit from focused clinical and research attention. Novel primary care interventions focusing on the practicalities of taking medicines are warranted.

Abstract word count- 298

Keywords: Stroke, Medication Adherence, Caregivers, Barthel

Article summary

Strengths and Limitations

- Development of the questionnaire was based on patients' and caregivers' own views gathered through workshops.
- Stroke survivors were recruited from two UK regions.
- This work identified issues from a population that includes patients severely affected by stroke, who are often excluded from research.
- Results shed light on the effect of stroke related impairments on practical domains and predictors of medicine taking, which have significant effects on medication adherence and call for new primary care interventions.
- The low response rate reported is a limitation of this study and stroke survivors who are harder to reach may have been missed.

Funding: This study was funded by the RCGP SFB, Ref. SFB 2014 – 15 'Quantifying the support stroke survivors get with daily medication taking: a questionnaire survey'. Anna De Simoni and Luis Ayerbe are funded by a NIHR Academic Clinical Lectureships. This article therefore presents independent research funded by NIHR. The views expressed are those of the authors and not necessarily those of the NHS, the NIHR, or the Department of Health. James Jamison was supported by a research grant from The Stroke Association and the British Heart Foundation: TSA BHF 2011/01

For peer review only - http://bmjopen.bmj.com/site/about/guidelines.xhtml

For peer review only

Data sharing statement: No additional data available.

Introduction

Stroke is the leading cause of disability in developed countries, with an estimated that 25-74% of the 50 million stroke survivors worldwide requiring some assistance or being fully dependent on caregivers for activities of daily living (ADLs).[1-3] For many older adults remaining independent at home may depend on how well they can manage complex medication regimens.[4 5] Around half of stroke survivors are dependent on others for everyday activities.[6]

There is evidence that being dependent for ADLs and impairment in mobility and communication decrease medication adherence in patients suffering from hypertension.[7] Deficits in attention, cognition or working memory have been linked with non-adherence to medications in other patient groups.[8] In a recent systematic review of medication adherence among patients with cognitive impairment, one third of studies showed that such patients were likely to have a caregiver to assist with medications and there was an association between taking four or more medicines and nonadherence.[9] In patients taking cardiovascular medicines, multiple factors including cognitive problems, lack of social support, dosing regimen, as well as practical problems and difficulties accessing services, contribute to poor medication adherence.[10 11] Low adherence to secondary prevention medication is associated with poor cardiovascular health.[12 13]

Stroke survivors have previously reported difficulties in the handling of medication as a barrier to adherence to secondary prevention medication after stroke.[14] This was true irrespective of age at stroke, with younger and older stroke survivors being similarly affected.[14] Research on medication adherence in stroke has identified multiple barriers to medication taking among stroke survivors.[14-16] However interventions developed to improve adherence have mainly concentrated on patients responsible for their own medicine taking.[17 18]

In England, the average age at stroke is 74 for men and 80 years for women.[19] In elderly patients in particular, cognitive deficits, taking large number of medicines and the complexity of medication regimens have been identified as barriers to medication adherence. [20 21] Caregivers are known to play a key role in

For peer review only - http://bmjopen.bmj.com/site/about/guidelines.xhtml

providing assistance to older people in a range of daily activities including medication taking and physician visits,[22] and can help improve adherence in cardiac patients with memory problems.[23]

Survivors of stroke have previously reported unmet needs including physical difficulties, cognitive and emotional difficulties, information needs and other unmet needs.[24 25] However we know little about factors that influence medication taking among stroke survivors with disabilities (i.e. physical or cognitive) living in the community (i.e. not in nursing homes), their unmet needs around the use of medicines or the proportion relying on caregivers for some or all aspects of medicine taking.

To date, survey instruments examining the unmet needs of stroke survivors have not focused on practical aspects of medication taking such as how patients collect or handle their medicines.

The aims of this investigation were to design an instrument to evaluate the support stroke survivors get with taking their medicines, characterise patients receiving help with medications, estimate the proportion who have unmet needs with daily medicine taking and who miss medications. We additionally aimed to identify the predictors of missing medicines and of experiencing unmet needs with medications.

This knowledge can inform the development of primary care interventions aimed at improving medication taking in this patients' group.

Methods

Questionnaire development workshops

To develop the questionnaire, current literature evidence was evaluated [17] and three workshops were conducted with 26 stroke survivors and 12 caregivers in the East of England (St John's College, Cambridge 2009 [26]: 7 patients, 1 caregiver; Different Strokes, Cambridge 2012: 9 patients, 3 caregivers; Peterborough, 2012: 10 patients, 8 caregivers). Recruitment was opportunistic and no purposive sampling was applied. The workshops were organised in the context of gathering Patient and Public Involvement (PPI) input into research grant applications aimed at improving adherence to medication after stroke.[17]

The survey questions were developed through thematic analysis [27] of workshops field notes.

For peer review only - http://bmjopen.bmj.com/site/about/guidelines.xhtml

A fourth workshop was conducted to gather feedback on the questionnaire using a PPI (Patient and Public Involvement) exercise with 11 stroke survivors and 3 caregivers recruited through a local stroke group (Different Strokes, East of England). Two stroke survivors from this group took part in subsequent 'think-aloud' interviews, which involved talking out loud as they read the questionnaire, continually verbalising what they were thinking.

Postal survey

In respect to sample size, 400 returned questionnaires would allow good precision for prevalence estimates. The 95% confidence intervals on various proportions with this sample size were calculated using the Wilson score method (with continuity correction) and are as follows: 50% (45.00-55.00%), 25% (20.89%-29.60%), 5% (3.16-7.74%). With 600 questionnaires, the improvement in the precision of the estimates would be as follows: 45.93%-54.07%, 21.62-28.70% and 3.46%-7.14% respectively.

General practices in primary care in the East of England and London were approached through the Clinical Research Network (CRN). CRN Eastern contacted 20 GP practices, of which 11 replied and took part in the study. CRN North London contacted 140 GP East London practices by email (Tower Hamlets, Newham and City & Hackney CCGs), of which only two replied and participated in the study. Five of the eight GP practices contacted in North London (Barnet CCG) through a research coordinator took part in the study.

Patients with stroke and their caregivers were sent the postal questionnaire according to the following criteria

Inclusion Criteria

Patients:

All patients aged > 18 on the practice stroke register with documented history of stroke.

Caregivers:

- Anyone identified by the patient as having a role helping with medicine taking.

Exclusion Criteria

- Patients who suffered a Transient ischaemic attack (TIA) but not a stroke.
- Palliative or end of life patients.

For peer review only - http://bmjopen.bmj.com/site/about/guidelines.xhtml

- Patients receiving institutional long term care (receiving total care in residential homes or living in nursing homes).
- Patients considered unsuitable to taking part in the study by their GP.

Survey participant identification

A list of prospective patients was compiled from the stroke register of each surgery by the practice staff. No restriction was placed on the recruitment of survivors who were dependent for ADLs or lacking capacity. The list was screened by a practice GP and anyone not meeting the inclusion criteria or who was considered unsuitable for the study was excluded.

Survey participant recruitment

Eligible participants were sent a study survey pack by practice staff between September 2016 and February 2017. Study recruitment packs included two invitation letters, information sheets, questionnaires and postal version of Barthel Index[28], one of which was for completion by the patient and the other by the caregiver. The Barthel Index provides a measure of functional independence and physical functioning and has been used in stroke research previously.[29] Patients with Barthel score 20 were categorised as independent for ADLs, those with score 15-19 moderately dependent for ADLs, and those with scores 0-14 severely dependent. [30] If receiving help with medications, the patient was asked to pass to their caregiver the invitation letter and information sheet and invite him/her to complete their copy of the questionnaire, providing answers on the patient's medicine taking. Family members, friends or paid caregivers of stroke survivors who were severely disabled and/or lacked mental capacity were invited to fill and return the caregivers' questionnaires only on behalf of patients. The information sheets stated that consent was implied by returning the completed questionnaire. Participants were asked to return completed questionnaires to the research centre in the FREEPOST envelopes provided. A second mail out of the study invitation pack was sent to all patients as a reminder, 2 weeks after the first one.

For peer review only - http://bmjopen.bmj.com/site/about/guidelines.xhtml

Ethical approval

This study has received ethical approval from Cambridge Central Research Ethics Committee (REC reference: 16/EE/0182) and from the Health Research Authority (IRAS project ID: 170931)

Survey Analysis

Survey data entry was performed by Document Capture Company.[31] Individual patients' characteristics (age, gender, time since stoke, number of daily medicines) were collected from the questionnaires themselves. Practice population, number of patients on stroke registers, deprivation score and ethnicity were taken from the National General Practice profiles (https://fingertips.phe.org.uk/profile/general-practice). The proportions of patients in each sociodemographic category, needing help taking medication, missing any medication in the previous 30 days, and reporting the need for more help taking medication, were estimated. When the survivor and caregiver questionnaires were both returned together, study data were collected from the patient's questionnaire only. The associations between 'Unmet needs' and age (< or ≥70 years), gender, total number of medicines taken, dependence for ADLs, years since stroke, and receiving help with medicines were investigated with individual logistic regression models (a different model per variable investigated), adjusted each and all of them for age and gender. Individual logistic regression models adjusted for age and gender were also used to estimate the association between 'Missed medicines in the previous 30 days' and age (< or > 70 years), gender, total number of medicines taken, dependent for ADLs, years since stroke, help with medicines and unmet needs (a different model adjusted for age and gender per variable investigated).

Sensitivity analysis was conducted to investigate if predictors of missing medication or unmet needs vary when the analysis was done on the whole dataset *versus* on questionnaires filled by patients only.

Chi squared tests were used to compare the responses on unmet needs and missing medication given by patients *versus* caregivers.

All statistical analysis has been conducted with Stata (version 14, StataCorp LP, College Station, TX, USA, 2013).

For peer review only - http://bmjopen.bmj.com/site/about/guidelines.xhtml

Results

Questionnaire development

Taking medications emerged as an important issue in all three workshops: nearly half of patients stated that a family member or friend was supporting them with daily medicine routines especially in relation to prompting medicine taking. This was put down to effects of the stroke itself on memory retention rather than general memory problems that people without stroke also experience. They admitted missing doses due to forgetting. Only a small proportion of survivors were actually handling their own prescriptions and were relying on support from family and/or community services. In one workshop almost all survivors had Dosette medication boxes and agreed that taking medications out of safety bottles and blister packs was a problem due to physical disabilities.

Thematic analysis of workshop data revealed five main practical domains of support needed with medication taking: 1) Dealing with prescriptions and collection of medicines; 2) Getting medicines out of the box, blister packs of bottles; 3) Prompting 'It's time to take your medicine'; 4) Swallowing medicines; and 5) Checking whether medicines have been taken. The final study questionnaire (see Supplementary file 1) included questions relating to each of these five domains, one item related to adherence (missed medicine in the last 30 days) and an assessment of disabilities through completion of the validated postal version of the Barthel Index.[28] The questionnaire was adapted for caregivers (see Supplementary file 2).

Questionnaire finalisation

On the basis of the fourth workshop and two 'think-aloud' interviews, we reworded the survey questions (e.g. from 'Do you get help with' was changed into 'Is somebody helping you with') and used a scale response 'All the time', 'Often', 'Sometimes', 'Rarely', 'Never' for the first question of each of the five survey domains, which was originally conceived as a 'yes' or 'no' answer (see supplementary file for text of questions).

For peer review only - http://bmjopen.bmj.com/site/about/guidelines.xhtml

Survey

Practice characteristics

18 GP practices agreed to take part in the study, of which just over 1/3 were in London (n=7). GP practices were relatively large with an average population of 11,904 patients (SD = 4010) and a low to moderate level of deprivation (Index of Multiple Deprivation[32] (IMD): Mean-7.05: SD-3.19). Out of 3066 patients on the stroke registers, 1687 stroke patients (55%) were considered eligible for the study and received the postal questionnaire. The average response rate of East of England and London practices was 42% and 27% respectively. The response rate varied between 16% and 53% across practices.

Participant characteristics

596 participants returned a completed questionnaire [549 (92.1 %) from patients, 47 (7.9 %) from caregivers showing a mean response rate of 35% (0.33-0.37). Participants were on average 72.7 yrs old. 37.8% (n=210) of the sample were female, see table 1. There were a high proportion of white patients in the recruited practices (79%), which were on average 21% of mixed or ethnic minority background. Approximately 28% of study participants were completely independent for ADLs.

Participants getting any kind of help with medicines were on average 73.6 years old, two thirds were male with only 19% of this group completely independent for ADLs.

Patients with unmet needs were on average 69 years old, predominantly male (71%) and 56.86% were severely dependent for ADLs. Patients who missed medications were on average 70 years old, 64% were male and the majority (48%) were moderately dependent for ADLs.

Table 1 here

For peer review only - http://bmjopen.bmj.com/site/about/guidelines.xhtml

Support with daily medication taking

Overall, 55.5% (95% CI: 51.7-59.7) of the participants received help in at least one aspect of taking medication, in that they ticked one of the options from 'all the time' to 'rarely' on one or more of the five questions related to medicine taking. 11% (95% CI: 8.8-13.9) of patients reported experiencing unmet needs and needing more help with at least one of the aspects of taking medication, in that they ticked 'yes' to the question "do you feel you need more help", on one or more of the five questions related to medicine taking. The proportion of questionnaires reporting unmet needs filled in by caregivers, 19.6% (n=9), and by patients, 10.7% (n=57), had no significant difference (p=0.068).

Among participants help was needed to some degree with prescriptions and collection of medicines (49.8 %), getting medicines out of the box or packet (27.9 %), reminding to take medicines (36.4 %), swallowing medicines (20.2 %) and checking that medicines have been taken (34.1 %).(see Table 2). Being reminded to take medicines, dealing with prescriptions and collection of medicines and getting medicines out of a pack or bottle were the most commonly reported areas of unmet needs. Almost two thirds of participants (65.3%) reported never missing medicines in the last 30 days. Out of the 34.7% of patients who said they missed taking medicine at any point in the previous 30 days, 23.9% said rarely, 9.3% sometimes, 0.8% often and 0.7% all the time. The proportion of questionnaires reporting missing medication at some point, filled in by caregivers, 27.7% (n=13), and by patients 35.3% (n=193), had no significant difference (p=0.292).

Table 2 here

Factors associated with unmet needs

Being on a higher total number of daily medications (OR: 1.2, (1.1-1.3), p<0.001), severe dependence for ADLs (OR: 11.62 (4.16-32.43) p<0.001) and receiving any kind of help (OR: 5.6, (2.7-11.63), p<0.001) in relation to taking medication was associated with experiencing unmet needs. Getting help with swallowing medicines (OR: 6.8, (3.8-12.02), p<0.001), getting medicines out of a box, blister packs or bottles (OR: 6.5, (3.6-11.8), p<0.001) showed the strongest associations with experiencing unmet needs (see table 3).

For peer review only - http://bmjopen.bmj.com/site/about/guidelines.xhtml

When the analyses were conducted with data from questionnaires filled by patients only, the variables significantly associated with unmet needs were the same, apart from years since stroke (Supplementary Appendix 1).

Table 3 here

Factors associated with missing medications

Being older (age ≥70) was associated with a lower probability of missing medication (OR: 0.59 (0.41-0.86) p=0.006). Being on a higher number of daily medicines (polypharmacy) (OR:1.07 (1.02-1.12), p=0.008) and getting any kind of help with medicine taking (OR:2.08 (1.43-3.03) p<0.001) was associated with higher probability of missing medicines. The more unmet needs stroke survivors had with taking medication, the more likely they were to miss their medicines (OR: 5.3 (3.0-9.5), p<0.001). (see Table 4). When the analyses were conducted with data from questionnaires filled by patients only, the variables significantly associated with missing medication were the same (Supplementary Appendix 1).

Table 4 here

Discussion

Summary of findings

From workshops we identified 5 key issues that patients regarded as important with medication taking after stroke. We converted these into a five item questionnaire that we distributed to people on stroke registers in 18 general practices. We obtained a response rate of 35%. Among respondents, 56% of survivors in the community were receiving help in some aspect of daily medication taking, 11% reported needing more help in

For peer review only - http://bmjopen.bmj.com/site/about/guidelines.xhtml

at least one domain of medicine taking and 34% missed taking their medicines at some point in the previous 30 days.

A higher total number of daily medicines, being severely dependent for ADLs and receiving help with medication were predictors of experiencing at least one unmet need in respect of medication taking. Stroke survivors who were younger, taking a higher number of daily medicines and experiencing a greater number of unmet needs were more likely to miss medications.

This work identified issues from a population that includes patients severely affected by stroke, who are often excluded from research.[17] Results presented here shed light on the effect of stroke related impairments on practical domains and predictors of medicine taking, which are shown to have significant effects on overall adherence.

Strengths and limitations

A strength of this study is that the questionnaire was developed from patients' and caregivers' own views gathered through workshops. Although not recruited through purposive sampling, workshop participants suffered from a range of stroke related impairments, as highlighted by the reported use of Dossette boxes, dependence on others for aspects of medicine taking like prompting medication times, and dependence for ADLs such as collecting prescriptions and taking tablets out of boxes. In the postal survey, the inclusion of stroke survivors regardless of level of dependence for ADLs permitted investigating a population who are understudied,[17] yet may have significant unmet needs that can affect their adherence to medications. This investigation highlights caregivers' role in managing medicines in survivors dependent for ADLs.

However, study limitations should also be considered. The response rate across recruited GP practices was low and harder to reach stroke survivors may have been missed. Poor response rate is a source of bias that might affect our estimates.

Interestingly, considering the average age at stroke in England (i.e. 74 for men and 80 years for women[19]), our participants' population was slightly younger (73 years), perhaps reflecting the fact that patients receiving institutional long term care were excluded from the study or that older people found harder taking part in a postal survey. Through the Barthel score, we did not assess cognition directly, although low cognitive function

For peer review only - http://bmjopen.bmj.com/site/about/guidelines.xhtml

For peer review only

is associated with poor adherence.[33] As the Barthel focuses on physical disability it is not known to what extent study participants were cognitively impaired or suffered from communication difficulties like aphasia. In addition, dependency for ADLs could have been caused by existing co-morbidities other than stroke. We did not collect information on the use of blister packaged medication or devices to aid compliance, which could have influenced medication taking practices. Finally this study examined all medicine taking and did not differentiate between stroke secondary prevention medications and other drug categories.

Comparisons with existing research

To our knowledge this is the first study that shows that more than half of all stroke survivors get help with some aspect of medicine taking and that those receiving help are more likely to have unmet needs. This provides some insight in to why adherence to medication in stroke survivors may be poor.[34]

Moreover, the greater the number of medicines, the more likely stroke survivors were to miss medications. Addressing pill burden by simplifying drug regimens may be an important focus for future interventions. Indeed the polypill approach to medication taking has been shown to reduce cardiovascular as well as total pill burden in a primary care setting.[35] Simpler dosing regimens, are known to be associated with better medication adherence [36] while fewer medicines has been shown to be an independent predictor of long term medication persistence among stroke survivors.[37 38] A recent trial incorporating a fixed-dose combination polypill approach to taking cardiovascular medicine demonstrated better adherence among patients receiving a single pill.[39]

Receiving help with prescriptions and collecting medicines was identified as the area where most help was received (49.7% of respondents). Stroke survivors who are dependent for activities of daily living may face considerable practical challenges accessing health care resources at the pharmacy and the GP practice. A recent study in the USA found that around 2/3 of caregivers were involved in at least 1 medication management activity of elderly patients and that high involvement in Instrumental Activities of Daily Living (IADLs) was associated with the caregiver providing the patient with assistance in ordering medicines.[40] Filling prescriptions is also known to be an important factor influencing medication adherence.[41 42] Indeed caregivers can play a significant role in ensuring appropriate medication taking. A recent interview study exploring potential barriers and facilitators of medication adherence in stroke identified the central role of the caregiver in medication adherence.[43] Our evaluation of an online stroke forum also confirmed the important

For peer review only - http://bmjopen.bmj.com/site/about/guidelines.xhtml

role of the caregiver in facilitating medication adherence.[14] Monitoring prescription collections, liaising with the GP and pharmacy, increasing the time between prescriptions or arranging medication deliveries, may help to address prescription needs.

Around 11% of stroke survivors reported unmet medication needs. We found that stroke survivors severely dependent for ADLs and receiving help with medicines were more likely to report unmet needs, which is in line with a recent study investigating stroke/TIA survivors in Australia, where greater functional ability was associated with fewer unmet needs, including those related to secondary prevention.[44] In previous research on unmet needs among stroke survivors, a 44 item survey study by McKevitt and colleagues (2011) reported that 49% of stroke survivors had at least one unmet need [25], while in a study of Australian survivors who completed a 58 item survey, the percentage was 84%.[24] Both these studies however examined unmet needs over a variety of domains including health, work, leisure and everyday living, social support and finances, whereas our study focused on medication needs only.

Getting help to take medicines out of a box, packet or bottle was the area where the greatest proportion of stroke survivors needed help all of the time. We previously found that the use of pill boxes and blister packed medication to be both a facilitator [35] and a barrier [14] to adherence among stroke survivors[15], while interventions using blister packaging and pill boxes have been found to be associated with improved adherence.[45] Although electronic medication devices were considered potentially effective in improving medication taking behaviour among patients with cognitive impairments, success in using such devices was dependent on the patient having a good level of dexterity, while removing the medication from these devices was also found to be challenging. [46 47 48]

The need for further support in this domain, as reported in the current study, suggests that handling medications remains problematic for stroke survivors.

An interesting finding from this survey study is that stroke survivors who missed medicines were younger. This is consistent with other research on adherence in stroke that found that younger age was predictive of poor adherence[49], and has also been described in patients taking medication for cardiovascular disease.[50] The finding in the present study contrasts with the view that older patients are more likely to face difficulties taking medication[51 52] which is frequently attributed to higher number of pre-existing comorbidities resulting in polypharmacy and increased complexity of medication taking regimens. The fact that older

For peer review only - http://bmjopen.bmj.com/site/about/guidelines.xhtml

patients may be less likely miss medicine might be down to the support they receive from caregivers. Our findings suggests that support needed with medications may be overlooked in younger stroke survivors.[53]

In this study a significant proportion of patients admitted missing medications occasionally. There is evidence that improving adherence by one anti-hypertensive pill/week for a once-a-day regimen reduces the hazard of stroke by 8–9 % and death by 7 %.[54] Each incremental 25% increase in proportion of days covered with statin medications is associated with a 0.10 mmol/L reduction in LDL-C cholesterol.[55 56] Non-adherence to cardiovascular medications is associated with increased risk of morbidity and mortality.[57]

Implications for clinical practice

A significant proportion of patients, particularly those who take large numbers of tablets, are disabled or receive help to take medication, have unmet needs and miss their tablets, which can increase risk of recurrent cardiovascular events. These particularly vulnerable groups of patients might benefit from focused clinical attention. Through understanding the needs of survivors and caregivers in different aspects of daily medication taking, we can help direct future resources to the areas of greatest need. For example, further exploration of medication packaging is warranted to understand the difficulties stroke survivors face handling medicines. Polypharmacy remains a difficulty for older patients. Therefore, exploring the use of combination pills and further efforts to reduce the burden of multiple medications among stroke survivors is warranted.

The questionnaire we have developed could be used to understand the challenges around medication faced by other patient groups. Unmet medication needs among UK stroke survivors have not been previously explored in the context of activities both survivors and caregivers consider important for taking medicines. Through understanding the extent of unmet needs as well as the areas in which these are greatest, strategies can be developed which address poor medication taking practices and therefore improve medication adherence.

Future research

Novel interventions focussing on the practicalities of taking medicines and aimed at improving stroke survivors' adherence to treatment are needed. The findings reported here may inform the development of such

For peer review only - http://bmjopen.bmj.com/site/about/guidelines.xhtml

interventions. Advances in technology have the potential to facilitate delivery of such interventions, e.g. electronic devices prompting medication taking times.[58 59] Efforts to improve medication taking among survivors of stroke using technology are already underway and have shown promise.[60].

Acknowledgements

The authors wish to thank all the stroke survivors and caregivers who participated in this study.

Competing interests

None declared

Data sharing statement

No additional data are available

Author/s contribution

ADS is the Chief Investigator, contributed to the study design, data analysis and commented on the manuscript. JJ contributed to the study design, data collection, data analysis and prepared the manuscript for submission. JM is a co-investigator on the study, wrote and commented on the manuscript. SS is a co-investigator on the study, wrote and commented on the manuscript. LA contributed to the data analysis and commented on the manuscript. GDiT contributed to the data analysis and commented on the manuscript. All authors agreed on the final draft of the submitted manuscript.

For peer review only

References

1. Young J, Forster A. Review of stroke rehabilitation. *BMJ* 2007;334(7584):86-90. doi: 10.1136/bmj.39059.456794.68
2. Miller EL, Murray L, Richards L, et al. Comprehensive overview of nursing and interdisciplinary rehabilitation care of the stroke patient: a scientific statement from the American Heart Association. *Stroke* 2010 (Oct);41(10):2402-48. doi: 10.1161/STR.0b013e3181e7512b
3. Feigin VL, Barker-Collo S, Parag V, et al. Auckland Stroke Outcomes Study: Part 1: Gender, stroke types, ethnicity, and functional outcomes 5 years poststroke. *Neurology* 2010;75(18):1597-607. doi: 10.1212/WNL.0b013e3181fb44b3
4. Marek KD AL. Medication Management of the Community-Dwelling Older Adult. In: RG H, ed. An Evidence-Based Handbook for Nurses: Agency for Healthcare Research and Quality (US). Rockville, MD2008.
5. Lewis A. Non-compliance: A $100 billion problem. . *The Remington Report* 1997;5:14-5.
6. National Audit Office. Progress in improving stroke care. In: Office NA, ed. London: Department of Health, 2010.
7. Park JH, Park JH, Lee SY, et al. Disparities in Antihypertensive Medication Adherence in Persons With Disabilities and Without Disabilities: Results of a Korean Population-Based Study. *Archives of Physical Medicine and Rehabilitation* 2008;89(8):1460-67. doi: http://dx.doi.org/10.1016/j.apmr.2007.12.045
8. Stilley CS, Bender CM, Dunbar-Jacob J, et al. The Impact of Cognitive Function on Medication Management: Three Studies. *Health psychology : official journal of the Division of Health Psychology, American Psychological Association* 2010;29(1):50-55. doi: 10.1037/a0016940
9. Smith D, Lovell J, Weller C, et al. A systematic review of medication non-adherence in persons with dementia or cognitive impairment. *PLoS ONE* 2017;12(2):e0170651. doi: 10.1371/journal.pone.0170651
10. Kronish IM, Ye S. Adherence to Cardiovascular Medications: Lessons Learned and Future Directions. *Progress in cardiovascular diseases* 2013;55(6):590-600. doi: 10.1016/j.pcad.2013.02.001

For peer review only

11. Gordon K, Smith F, Dhillon S. Effective chronic disease management: Patients' perspectives on medication-related problems. *Patient Education and Counseling* 2007;65(3):407-15. doi: http://dx.doi.org/10.1016/j.pec.2006.09.012

12. Xu J, Zhao X, Wang Y, et al. Impact of a Better Persistence with Antihypertensive Agents on Ischemic Stroke Outcomes for Secondary Prevention. *PLoS ONE* 2013;8(6):e65233. doi: 10.1371/journal.pone.0065233

13. Colivicchi F, Bassi A, Santini M, et al. Discontinuation of Statin Therapy and Clinical Outcome After Ischemic Stroke. *Stroke* 2007;38(10):2652-57. doi: 10.1161/strokeaha.107.487017

14. Jamison J, Sutton S, Mant J, et al. Barriers and facilitators to adherence to secondary stroke prevention medications after stroke: Analysis of survivors and caregivers views from an online stroke forum. *BMJ Open* 2017;7 (e016814)

15. Jamison J, Graffy J, Mullis R, et al. Barriers to medication adherence for the secondary prevention of stroke: a qualitative interview study in primary care. *The British Journal of General Practice* 2016;66(649):e568-e76. doi: 10.3399/bjgp16X685609

16. Souter C, Kinnear A, Kinnear M, et al. Optimisation of secondary prevention of stroke: a qualitative study of stroke patients' beliefs, concerns and difficulties with their medicines. *International Journal of Pharmacy Practice* 2014;22(6):424-32. doi: 10.1111/ijpp.12104

17. De Simoni A, Mant J, Sutton S. Adherence to medication in stroke survivors dependent on caregivers. *British Journal of General Practice* 2015;65(640):e789.

18. De Simoni A, Hardeman W, Mant J, et al. Trials to Improve Blood Pressure Through Adherence to Antihypertensives in Stroke/TIA: Systematic Review and Meta-Analysis. *Journal of the American Heart Association* 2013 (Aug 20);2(4):e000251. doi: 10.1161/jaha.113.000251

19. Royal College of Physicians. Sentinel Stroke National Audit Programme (SSNAP). National clinical audit annual results portfolio March 2015-April 2016. [Available from: http://bit.ly/1M5R3Op

20. Munger MA, Van Tassell BW, LaFleur J. Medication Nonadherence: An Unrecognized Cardiovascular Risk Factor. *Medscape General Medicine* 2007;9(3):58-58.

21. Brown MT, Bussell JK. Medication Adherence: WHO Cares? *Mayo Clinic proceedings* 2011;86(4):304-14. doi: 10.4065/mcp.2010.0575

For peer review only - http://bmjopen.bmj.com/site/about/guidelines.xhtml

22. Wolff JL, Spillman B. Older Adults Receiving Assistance With Physician Visits and Prescribed Medications and Their Family Caregivers: Prevalence, Characteristics, and Hours of Care. *The Journals of Gerontology: Series B* 2014;69(Suppl_1):S65-S72. doi: 10.1093/geronb/gbu119

23. Ownby RL, Hertzog C, Crocco E, et al. Factors related to medication adherence in memory disorder clinic patients. *Aging & mental health* 2006;10(4):378-85. doi: 10.1080/13607860500410011

24. Andrew NE, Kilkenny M, Naylor R, et al. Understanding Long-Term Unmet Needs in Australian Survivors of Stroke. *International Journal of Stroke* 2014;9(SA100):106-12. doi: doi:10.1111/ijs.12325

25. McKevitt C, Fudge N, Redfern J, et al. Self-Reported Long-Term Needs After Stroke. *Stroke* 2011;42(5):1398-403. doi: 10.1161/strokeaha.110.598839

26. De Simoni A, Kellar I, Sutton S, et al. Developing a pharmacist intervention to improve adherence to hypertensive medications in TIA/stroke patients. SAPC Conference. Norwich, 2010.

27. Braun V, Clarke V. Using thematic analysis in psychology. *Qualitative Research in Psychology* 2006;3(2):77-101. doi: 10.1191/1478088706qp063oa

28. Gompertz P, Pound P, Ebrahim S. A postal version of the Barthel Index. *Clinical Rehabilitation* 1994;8(3):233-39. doi: doi:10.1177/026921559400800308

29. Quinn TJ, Langhorne P, Stott DJ. Barthel Index for Stroke Trials. *Development, Properties, and Application* 2011;42(4):1146-51. doi: 10.1161/strokeaha.110.598540

30. Wolfe CD, Taub NA, Woodrow EJ, et al. Assessment of scales of disability and handicap for stroke patients. *Stroke* 1991;22(10):1242-44. doi: 10.1161/01.str.22.10.1242

31. Document Capture Company [Available from: http://www.documentcapture.co.uk/data-collection-methods/

32. Smith T, Noble, M, Noble S, et al. The English Indices of Deprivation 2015 Research report, 2015.

33. Hayes TL, Larimer N, Adami A, et al. Medication Adherence in Healthy Elders. *Journal of Aging and Health* 2009;21(4):567-80. doi: 10.1177/0898264309332836

34. Chowdhury R, Khan H, Heydon E, et al. Adherence to cardiovascular therapy: a meta-analysis of prevalence and clinical consequences. *European Heart Journal* 2013;34:2940-8. doi: 10.1093/eurheartj/eht295

For peer review only

35. Truelove M, Patel A, Bompoint S, et al. The Effect of a Cardiovascular Polypill Strategy on Pill Burden. *Cardiovascular Therapeutics* 2015;33(6):347-52. doi: 10.1111/1755-5922.12151

36. Claxton AJ, Cramer, J., Pierce, C. A Systematic Review of the Associations Between Dose Regimens and Medication Compliance *Clinical therapeutics* 2001;23(8):1296-310.

37. Bushnell CD, Olson DM, Zhao X, et al. Secondary preventive medication persistence and adherence 1 year after stroke. *Neurology* 2011;77(12):1182-90. doi: 10.1212/WNL.0b013e31822f0423 [published Online First: 2011/09/09]

38. Chapman RH, Benner JS, Petrilla AA, et al. Predictors of adherence with antihypertensive and lipid-lowering therapy. *Archives of Internal Medicine* 2005;165(10):1147-52. doi: 10.1001/archinte.165.10.1147

39. Thom S, Poulter N, Field J, et al. Effects of a fixed-dose combination strategy on adherence and risk factors in patients with or at high risk of CVD: the UMPIRE randomized clinical trial. *JAMA : the journal of the American Medical Association* 2013;310(9):918-29. doi: 10.1001/jama.2013.277064

40. Noureldin M, Plake KS. Correlates of caregivers' involvement in the management of older adults' medications. *Research in Social and Administrative Pharmacy* 2017;13(4):840-48. doi: https://doi.org/10.1016/j.sapharm.2016.09.009

41. Andersson K, Melander A, Svensson C, et al. Repeat prescriptions: refill adherence in relation to patient and prescriber characteristics, reimbursement level and type of medication. *European Journal of Public Health* 2005;15(6):621-26. doi: 10.1093/eurpub/cki053

42. Vlasnik JJ, Aliotta SL, DeLor B. Medication adherence: Factors influencing compliance with prescribed medication plans. *The Case Manager* 2005;16(2):47-51. doi: http://dx.doi.org/10.1016/j.casemgr.2005.01.009

43. Bauler S, Jacquin-Courtois S, Haesebaert J, et al. Barriers and Facilitators for Medication Adherence in Stroke Patients: A Qualitative Study Conducted in French Neurological Rehabilitation Units. *European Neurology* 2014;72(5-6):262-70.

44. Olaiya MT, Cadilhac DA, Kim J, et al. Long-term unmet needs and associated factors in stroke or TIA survivors: An observational study. *Neurology* 2017 (Jul 4);89(1):68-75. doi: 10.1212/wnl.0000000000004063

For peer review only

For peer review only - http://bmjopen.bmj.com/site/about/guidelines.xhtml

45. Conn VS, Ruppar TM, Chan KC, et al. Packaging interventions to increase medication adherence: systematic review and meta-analysis. *Current Medical Research and Opinion* 2015;31(1):145-60. doi: 10.1185/03007995.2014.978939

46. Hall J, Bond C, Kinnear M, et al. Views of patients and professionals about electronic multicompartment medication devices: a qualitative study. *BMJ Open* 2016;6:e012915. doi: 10.1136/bmjopen-2016-012915

47. Beckman AGK, Parker MG, Thorslund M. Can elderly people take their medicine? *Patient Education and Counseling* 2005;59(2):186-91. doi: http://dx.doi.org/10.1016/j.pec.2004.11.005

48. Notenboom K, Beers E, van Riet-Nales DA, et al. Practical Problems with Medication Use that Older People Experience: A Qualitative Study. *Journal of the American Geriatrics Society* 2014;62(12):2339-44. doi: 10.1111/jgs.13126

49. O'Carroll R, Whittaker J, Hamilton B, et al. Predictors of adherence to secondary preventive medication in stroke patients. *Ann Behav Med* 2011;41(3):383-90. doi: 10.1007/s12160-010-9257-6 [published Online First: 2011/01/05]

50. Crowley MJ, Zullig LL, Shah BR, et al. Medication Non-Adherence After Myocardial Infarction: An Exploration of Modifying Factors. *Journal of General Internal Medicine* 2015;30(1):83-90. doi: 10.1007/s11606-014-3072-x

51. Hughes CM. Medication Non-Adherence in the Elderly. *Drugs & Aging* 2004;21(12):793-811. doi: 10.2165/00002512-200421120-00004

52. De Schryver EL, van Gijn J, Kappelle LJ, et al. Non-adherence to aspirin or oral anticoagulants in secondary prevention after ischaemic stroke. *J Neurol* 2005;252(11):1316-21. doi: 10.1007/s00415-005-0858-0 [published Online First: 2005/05/04]

53. Kersten P, Low JTS, Ashburn A, et al. The unmet needs of young people who have had a stroke: results of a national UK survey. *Disability and Rehabilitation* 2002;24(16):860-66. doi: 10.1080/09638280210142167

54. Bailey JE, Wan JY, Tang J, et al. Antihypertensive medication adherence, ambulatory visits, and risk of stroke and death. *J Gen Intern Med* 2010;25(6):495-503. doi: 10.1007/s11606-009-1240-1 [published Online First: 2010/02/19]

For peer review only

For peer review only - http://bmjopen.bmj.com/site/about/guidelines.xhtml

55. Amarenco P, Labreuche J. Lipid management in the prevention of stroke: review and updated meta-analysis of statins for stroke prevention. *The Lancet Neurology* 2009;8(5):453-63. doi: 10.1016/s1474-4422(09)70058-4 [published Online First: 2009/04/21]

56. Ho PM, Rumsfeld JS, Masoudi FA, et al. Effect of medication nonadherence on hospitalization and mortality among patients with diabetes mellitus. *Arch Intern Med* 2006;166(17):1836-41. doi: 10.1001/archinte.166.17.1836 [published Online First: 2006/09/27]

57. Rasmussen JN, Chong A, Alter DA. Relationship between adherence to evidence-based pharmacotherapy and long-term mortality after acute myocardial infarction. *Jama* 2007;297(2):177-86. doi: 10.1001/jama.297.2.177 [published Online First: 2007/01/11]

58. Tao D, Xie L, Wang T, et al. A meta-analysis of the use of electronic reminders for patient adherence to medication in chronic disease care. *Journal of Telemedicine and Telecare* 2015;21(1):3-13. doi: doi:10.1177/1357633X14541041

59. Thakkar J, Kurup R, Laba T, et al. Mobile telephone text messaging for medication adherence in chronic disease: A meta-analysis. *JAMA Internal Medicine* 2016;176(3):340-49. doi: 10.1001/jamainternmed.2015.7667

60. Kamal AK, Shaikh Q, Pasha O, et al. A randomized controlled behavioral intervention trial to improve medication adherence in adult stroke patients with prescription tailored Short Messaging Service (SMS)-SMS4Stroke study. *BMC Neurology* 2015;15(1):212. doi: 10.1186/s12883-015-0471-5

For peer review only

	All patients				Patients who receive any kind of help				Patients with unmet needs				Patients who miss medication			
	N	%	Mean	SD	N	%	Mean	SD	N	%	Mean	SD	N	%	Mean	SD
Age	588		72.7	11.6	331		73.6	12.2	64		68.8		203		70.5	13.0
Female	210	37.8			112	36.2			18	28.6			68	35.6		
Male	346	67.2			197	63.2			45	71.4			123	64.4		
Time since stroke	535		7.7	7.6	295		7.97	8.5	61		9.3	9.2	186		7.7	8.5
N of daily medicines	557		6.4	4	312		7.3	4.5	59		9.7	7.1	190		6.9	4.1
Independent for ADLs (BI=20)	139	28.3			53	18.9			5	9.8			45	25.7		
Moderately dependent for ADLs (BI=15-19)	231	47.1			130	46.4			17	33.3			84	48.0		
Severely dependent for ADLs (BI=0-14)	121	24.6			97	34.6			29	56.9			46	26.3		

Table 1. Characteristics of participants who took part in the survey study *(mean scores reported unless otherwise stated).* N represents the number of participants who completed the survey in respect to the different variables. BI: Barthel Index.

For peer review only

	N	All the Time N *(%)*	Often N *(%)*	Sometimes N *(%)*	Rarely N *(%)*	Never N *(%)*	Yes N *(%)*	No N *(%)*
Question 1 Is somebody helping with prescriptions and collection of your medicines?	583	186 *(31.9)*	19 *(3.3)*	40 *(6.9)*	45 *(7.7)*	293 *(50.2)*		
Question 1a Do you feel you need more help with prescriptions and collection of your medicines?	551						33 *(6.0)*	518 *(94.0)*
Question 2 Is somebody helping you getting the medicines out of the box, bottle or blister pack?	578	85 *(14.7)*	15 *(2.6)*	31 *(5.4)*	30 *(5.2)*	417 *(72.1)*		
Question 2a Do you feel you need more help with getting the medicines out of the box, bottle or blister pack?	553						33 *(6.0)*	520 *(94.0)*
Question 3 Is somebody helping with reminding you when is the time to take your medicine?	577	78 *(13.6)*	22 *(3.8)*	59 *(10.2)*	51 *(8.8)*	367 *(63.6)*		
Question 3a Do you feel you need more help with reminding when is the time to take your medicine?	564						35 *(6.2)*	529 *(93.8)*
Question4 Is somebody helping you with swallowing your medicine?	579	56 *(9.7)*	11 *(1.9)*	29 *(5.0)*	21 *(3.6)*	462 *(79.8)*		
Question 4a Do you feel you need more help with swallowing your medicine?	560						9 *(1.6)*	551 *(98.4)*
Question 5 I somebody helping you with checking that you have taken your medicines	576	76 *(13.2)*	23 *(4.0)*	58 *(10.0)*	40 *(6.9)*	379 *(65.9)*		
Question 5 a Do you feel you need more help with checking that you have taken your medicine	558						20 *(3.6)*	538 *(96.4)*
Thinking of the last 30 days, how often did you miss taking your regular medicines?	594	4 *(0.7)*	5 *(0.8)*	55 *(9.3)*	142 *(23.9)*	388 *(65.3)*		

Table 2. Results summarising participants' responses to the survey questions.

For peer review only - http://bmjopen.bmj.com/site/about/guidelines.xhtml

	Univariable analysis		Multivariable analysis	
**Variable**	**N**	**Odds ratio (95% CI) p value**	**N**	**Odds ratio (95% CI) p value**
Age ≥70	581	0.6 (0.4-1.1) p=0.084	544	0.7 (0.4-1.2) p=0.180
Gender (female)	544	0.7 (0.4-1.2) p=0.137	544	0.7 (0.4-1.2) p=0.147
Number of different medicines	542	1.2 (1.1-1.3) p<0.001	509	1.2 (1.1-1.3) p<0.001
Moderate Dependence for ADLs (BI: 15-19)	479	2.2 (0.8-6.1) p=0.135	447	2.7 (1.0-7.5) p=0.068
Severe Dependence for ADLs (BI: 0-14)	479	8.5 (3.2-22.8) p<0.001	447	11.6 (4.2-32.4) p<0.001
Years since stroke	522	1.0 (1.0-1.1) p=0.078	490	1.0 (1.0-1.1) p=0.160
Getting help with prescriptions and collection of medication	568	4.7 (2.5-8.8) p<0.001	533	4.6 (2.4-8.7) p<0.001
Getting help with taking medicines out of the box, bottle or blister pack	563	6.7 (3.8-11.8) p<0.001	527	6.6 (3.6-11.8) p<0.001
Getting help with reminding you when is the time to take your medicine?	562	4.7 (2.7-8.2) p<0.001	526	4.3 (2.4-7.6) p<0.001
Getting help to swallow the medication	565	6.7 (3.9-11.6) p<0.001	528	6.8 (3.8-12.0) p<0.001
Getting help by checking that you have taken your medicines	562	4.9 (2.8-8.6) p<0.001	526	5.9 (3.1-10.1) p<0.001
Getting any kind of help	574	5.9 (2.8-12.1) p<0.001	537	5.9 (2.7-11.6) p<0.001

Table 3. Results of the multivariable analysis showing the variables associated with unmet needs.
N: number of observations; ADLs: Activities of daily living; BI: Barthel Index.

For peer review only

For peer review only - http://bmjopen.bmj.com/site/about/guidelines.xhtml

	Univariable analysis		Multivariable analysis	
**Variable**	**N**	**Odds Ratio (95% CI) p value**	**N**	**Odds Ratio (95% CI) p value**
Age ≥70	594	0.6 (0.4-0.8) p=0.003	555	0.6 (0.4-0.9) p=0.006
Gender (female)	555	0.9 (0.6-1.2) p=0.401	555	0.9 (0.6-1.3) p=0.498
Number of different medicines	555	1.0 (1.0-1.1) p=0.040	520	1.1 (1.0-1.1) p=0.008
Moderate Dependence for ADLs (BI: 15-19)	490	1.2 (0.8-1.8) p=0.468	456	1.3 (0.8-2.0) p=0.343
Severe dependence for ADLs (BI 0-14)	490	1.3 (0.8-2.1) p=0.342	456	1.4 (0.8-2.4) p=0.239
Years since stroke	533	1.0 (0.9-1.0 p=0.950	499	1.0 (0.9-1.0) p=0.971
Getting help with prescriptions and collection of medication	581	2.0 (1.5-2.9) p<0.001	544	2.3 (1.6-3.3) p<0.001
Getting help to have the medicines out of the box, bottle or blister pack	576	1.4 (1.0-2.0) p=0.089	538	1.5 (1.0-2.2) p=0.051
Getting help with reminding you when is the time to take your medicine?	575	2.5 (1.7-3.6) p<0.001	537	2.7 (1.8-3.9) p<0.001
Getting help to swallow the medication	578	1.5 (1.0-2.3) p=0.045	539	1.7 (1.1-2.6) p=0.022
Getting help by checking that you have taken your medicines	576	2.4 (1.7-3.4) p<0.001	537	2.5 (1.7-3.7) p<0.001
Getting any kind of help	587	2.1 (1.4-3.0) p<0.001	548	2.1 (1.4-3.0) p<0.001
Unmet needs (participant reported more help needed)	580	5.3 (3.0-9.2) p<0.000	544	5.3 (3.0-9.4) p<0.001

Table 4. Results of univariable and multivariable analysis showing associations with missing medicines.
N: number of observations; ADLs: Activities of daily living; BI: Barthel Index.

For peer review only

	Unmet needs		Missing medication	
**Variable**	**N**	**Odds ratio (95% CI) p value**	**N**	**Odds ratio (95%CI) p value**
Age ≥70	498	0.7 (0.4-1.2) p=0.248	508	0.6 (0.4-0.9) p=0.009
Gender (female)	498	0.7 (0.4-1.3) p=0.262	508	0.9 (0.6-1.3) p=0.576
Number of different medicines	465	1.2 (1.1-1.3) p<0.001	475	1.1 (1.0-1.1) p=0.015
Moderate Dependence for ADLs (BI: 15-19)	408	2.6 (0.9-7.5) p<0.075	417	0.3 (-0.2-0.7) p=0.289
Severe Dependence for ADLs (BI: 0-14)	408	10.9 (3.8-31.0) p<0.001	417	0.5 (-0.1-1.0) p=0.119
Years since stroke	446	1.0 (1.0-1.1) p=0.036	454	1.0 (0.9-1.03) p=0.725
Getting help with prescriptions and collection of medication	487	4.6 (2.4-8.9) p<0.001	497	2.6 (1.8-3.8) p<0.001
Getting help with taking medicines out of the box, bottle or blister pack	481	6.6 (3.6-12.2) p<0.001	491	1.7 (1.1-2.6) p=0.20
Getting help with reminding you when is the time to take your medicine?	480	4.7 (2.6-8.5) p<0.001	490	3.0 (2.0-4.5) p<0.001
Getting help to swallow the medication	482	7.8 (4.2-14.8) p<0.001	492	2.1 (1.3-3.4) p=0.005
Getting help by checking that you have taken your medicines	480	5.9 (3.2-10.9) p<0.001	490	2.8 (1.8-4.2) p<0.001
Getting any kind of help	491	5.6 (2.7-11.9) p<0.001	501	2.3 (1.6-3.4) p<0.001

Supplementary Appendix 1. Predictors of unmet needs and missing medication, responses given by patients only (i.e. caregivers' filled questionnaire excluded). Multivariable analyses, all models adjusted for age and gender. N: number of observations.

For peer review only

Queen Mary University of London

UNIVERSITY OF CAMBRIDGE

QUESTIONNAIRE – PATIENT

Many people with stroke need help with taking their medicines. We would like to ask you few questions about the help you might need with taking your medicines.

How many different type of medicines do you take in one day? ☐
Write the number or an approximation. Please count all medications including eye drops, injections etc.

How old are you? ☐ How long ago was your stroke? ☐ years

What is your sex? M F

For each question below, please tick the box that best describes how you have taken your medicines in the last month:

1. **Is somebody helping with prescriptions and collection of your medicines?**
   - all the time ☐
   - often ☐
   - sometimes ☐
   - rarely ☐
   - never ☐

   Do you feel you need more help with prescriptions and collection of your medicines?

   Yes ☐ **No** ☐

2. **Is somebody helping you getting the medicines out of the box, bottle or blister pack?**
   - all the time ☐
   - often ☐
   - sometimes ☐
   - rarely ☐
   - never ☐

   Do you feel you need more help with getting the medicines out of the box, bottle or blister pack?

   Yes ☐ **No** ☐

For peer review only

3. **Is somebody helping with reminding you when is the time to take your medicine?**

all the time ☐
often ☐
sometimes ☐
rarely ☐
never ☐

Do you feel you need more help with reminding when is the time to take your medicine?

Yes ☐ **No** ☐

4. **Is somebody helping you with swallowing your medicine? For example by giving you a drink**

all the time ☐
often ☐
sometimes ☐
rarely ☐
never ☐

Do you feel you need more help with swallowing your medicine?

Yes ☐ **No** ☐

5. **Is somebody helping you with checking that you have taken your medicines?**

all the time ☐
often ☐
sometimes ☐
rarely ☐
never ☐

Do you feel you need more help with checking that you have taken your medicine?

Yes ☐ **No** ☐

Missing medicines

Thinking of the last 30 days, how often did you miss taking your regular medicines?

all the time ☐
often ☐
sometimes ☐
rarely ☐
never ☐

Remember - tick one box only

For peer review only

Barthel Questionnaire

These are some questions about your ability to look after yourself.

They may not seem to apply to you.

Please answer them all.

Tick one box in each section.

Bathing

In the bath or shower do you:

Remember - tick one box only

- manage on your own? ☐
- need help getting in and out? ☐
- need other help? ☐
- never have a bath or shower? ☐
- need to be washed in bed? ☐

Stairs

Do you climb stairs at home:

Remember - tick one box only

- without any help? ☐
- with someone carrying your frame? ☐
- with someone encouraging you? ☐
- with physical help? ☐
- not at all? ☐
- don't have stairs? ☐

Dressing

Do you get dressed:

Remember - tick one box only

- without any help? ☐
- just with help with buttons? ☐
- with someone helping you most of the time? ☐

Mobility

Do you walk indoors:

Remember - tick one box only

- without any help apart from a frame? ☐
- with one person watching over you? ☐
- with one person helping you? ☐
- with more than one person helping? ☐
- not at all? ☐
- Or do you use a wheelchair independently? (e.g. round corners) ☐

Transfer

Do you move from bed to chair:

Remember - tick one box only

- on your own? ☐
- with a little help from one person? ☐
- with a lot of help from one or more people? ☐
- not at all? ☐

For peer review only

Feeding

Do you eat food:		
	without any help?	☐
	with help cutting food or spreading butter?	☐
Remember - tick one box only	with more help?	☐

Toilet use

Do you use a toilet or commode:		
	without any help?	☐
	with some help but can do something?	☐
Remember - tick one box only	with quite a lot of help?	☐

Grooming

Do you brush your hair and teeth Wash your face and shave:		
	without help?	☐
Remember - tick one box only	with help?	☐

Bladder

Are you incontinent of urine?		
	never	☐
	less than once a week	☐
Remember - tick one box only	less than once a day	☐
	more often	☐
	Or do you have a catheter managed for you	☐

Bowels

Do you soil yourself?		
	never	☐
	Occasional accident	☐
Remember - tick one box only	all the time	☐
	or do you need someone to give you an enema?	☐

For peer review only

□□.□□□

Help with taking tablets after stroke

FAMILY MEMBER/FRIEND OR PRIVATE CARER COPY

Many people with stroke need help with taking their medicines. We would like to ask you few questions about the help you might be offering to your family member/friend/ patient with stroke with taking medicines.

Relation with your family member/friend with stroke

Are you:

Remember - tick one box only

- partner □
- son or daughter □
- friend □
- carer from an agency □
- other □
- if other, please specify

How many different types of medicines does your family member/friend/patient with stroke take in one day? □
Write the number or an approximation. Please count all medications including eye drops, injections etc.

How old is your family member/friend/patient with stroke? (years) □

How many years ago was your family member/friend/patient's stroke? □

What is your family member/friend/patient with stroke sex? M □ F □

For each question below, please tick the box that best describes the help needed by your family member/friend/patient with stroke with taking medicines in the last month.

1. **Is somebody helping your family member/friend/patient with stroke with prescriptions and collection of his/her medicines?**

   - all the time □
   - often □
   - sometimes □
   - rarely □
   - never □

Do you feel your family member/friend/patient with stroke needs more help with prescriptions and collection of his/her medicines?

Yes □ **No** □

Version 1.0 010316

For peer review only - http://bmjopen.bmj.com/site/about/guidelines.xhtml

☐☐.☐☐☐

2. Is somebody helping your family member/friend/patient with stroke getting the medicines out of the box, bottle or blister pack?

all the time ☐
often ☐
sometimes ☐
rarely ☐
never ☐

Do you feel your family member/friend/patient with stroke needs more help with getting the medicines out of the box, bottle, or blister pack?

Yes ☐ **No** ☐

3. Is somebody helping your family member/friend/patient with stroke with reminding when is time to take his/her medicine?

all the time ☐
often ☐
sometimes ☐
rarely ☐
never ☐

Do you feel your family member/friend/patient with stroke needs more help with reminding when is time to take his/her medicine?

Yes ☐ **No** ☐

4. Is somebody helping your family member/friend/patient with stroke with swallowing his/her medicine? For example by giving a drink.

all the time ☐
often ☐
sometimes ☐
rarely ☐
never ☐

Do you feel you your family member/friend/patient with stroke need more help with swallowing his/her medicine?

Yes ☐ **No** ☐

5. Is somebody helping your family member/friend/patient with stroke with checking that he/she has taken his/her medicines?

all the time ☐
often ☐
sometimes ☐
rarely ☐
never ☐

Do you feel your family member/friend/patient with stroke needs more help with checking that he/she has taken his/her medicine?

Yes ☐ **No** ☐

Version 1.0 010316

For peer review only

☐☐.☐☐☐

Missing medicines

Thinking of the last 30 days, how often did your family member/friend/patient with stroke miss taking his/her regular medicines?

Remember - tick one box only

- all the time ☐
- often ☐
- sometimes ☐
- rarely ☐
- never ☐

Barthel Questionnaire

These are some questions about the ability of your family member/friend/patient with stroke to look after him/herself.

Please answer them all.

Please fill this questionnaire even if you are not regularly caring for your family member/friend/patient with stroke, trying to answer questions in the way you think most accurately describes the disability of your family member/friend/patient with stroke.

Tick one box in each section.

Bathing

In the bath or shower do you:

Remember - tick one box only

- manage on your own? ☐
- need help getting in and out? ☐
- need other help? ☐
- never have a bath or shower? ☐
- need to be washed in bed? ☐

Stairs

Do you climb stairs at home:

Remember - tick one box only

- without any help? ☐
- with someone carrying your frame? ☐
- with someone encouraging you? ☐
- with physical help? ☐
- not at all? ☐
- don't have stairs? ☐

Dressing

Do you get dressed:

Remember - tick one box only

- without any help? ☐
- just with help with buttons? ☐
- with someone helping you most of the time? ☐

Mobility

Do you walk indoors:

Remember - tick one box only

- without any help apart from a frame? ☐
- with one person watching over you? ☐
- with one person helping you? ☐

Version 1.0 010316

For peer review only

☐ ☐ . ☐ ☐ ☐

	with more than one person helping?	☐
	not at all?	☐
	Or do you use a wheelchair independently? (e.g. round corners)	☐

Transfer

Do you move from bed to chair:	on your own?	☐
	with a little help from one person?	☐
Remember - tick one box only	with a lot of help from one or more people?	☐
	not at all?	☐

Feeding

Do you eat food:	without any help?	☐
	with help cutting food or spreading butter?	☐
Remember - tick one box only	with more help?	☐

Toilet use

Do you use a toilet or commode:	without any help?	☐
	with some help but can do something?	☐
Remember - tick one box only	with quite a lot of help?	☐

Grooming

Do you brush your hair and teeth	without help?	☐
Wash your face and shave:	with help?	☐
Remember - tick one box only		

Bladder

Are you incontinent of urine?	never	☐
	less than once a week	☐
Remember - tick one box only	less than once a day	☐
	more often	☐
	Or do you have a catheter managed for you	☐

Bowels

Do you soil yourself?	never	☐
	Occasional accident	☐
Remember - tick one box only	all the time	☐
	or do you need someone to give you an enema?	☐

For peer review only

BMJ Open

Evaluating practical support stroke survivors get with medicines and unmet needs in primary care: A survey

Journal:	*BMJ Open*
Manuscript ID	bmjopen-2017-019874.R2
Article Type:	Research
Date Submitted by the Author:	23-Jan-2018
Complete List of Authors:	JAMISON, JAMES; UNIVERSITY OF CAMBRIDGE, PUBLIC HEALTH & PRIMARY CARE<br>Ayerbe, Luis; Queen Mary University of London, Centre for Primary Care and Public Health<br>Di Tanna, Gian Luca; Queen Mary University of London<br>Sutton, Stephen; University of Cambridge,<br>Mant, Jonathan; University of Cambridge, General Practice and Primary Care Research Unit<br>De Simoni, Anna; Queen Mary University of London, Centre for Primary Care and Public Health
**Primary Subject Heading**:	Cardiovascular medicine
Secondary Subject Heading:	Cardiovascular medicine, Public health, General practice / Family practice
Keywords:	Medication adherence, Caregivers, Barthel, STROKE MEDICINE

SCHOLARONE™
Manuscripts

Evaluating practical support stroke survivors get with medicines and unmet needs in primary care: A survey

*James Jamison[1], MSc

Luis Ayerbe[2], PhD

Gian Luca Di Tanna[2] PhD

Stephen Sutton[1], PhD

Jonathan Mant[1], MD

Anna De Simoni[2], PhD

[1]Primary Care Unit, Department of Public Health & Primary Care, Forvie Site, University of Cambridge School of Clinical Medicine, Box 113 Cambridge Biomedical Campus, Cambridge, CB2 0SR.

[2]Centre for Primary Care and Public Health, Barts and The London School of Medicine and Dentistry, Yvonne Carter Building, London E1 2AB.

*corresponding author: James Jamison; Tel: +44 (0)1223 768272. Fax: +44 (0)1223 763492. Email:

jj285@medschl.cam.ac.uk

For peer review only - http://bmjopen.bmj.com/site/about/guidelines.xhtml

Abstract

Objectives

To design a questionnaire and use it to explore unmet needs with practical aspects of medicines taking after stroke, predictors of medicine taking and to estimate the proportion of survivors who get support with daily medication taking.

Design

Four workshops with stroke survivors and caregivers to design the questionnaire.

A cross-sectional postal questionnaire in primary care.

Setting

18 GP practices in the East of England and London. Questionnaires posted between September 2016 and February 2017.

Participants

1687 stroke survivors living in the community outside institutional long term care.

Primary Outcome measures

The proportion of community stroke survivors receiving support from caregivers for practical aspects of medicine taking; the proportion with unmet needs in this respect; the predictors of experiencing unmet needs and missing taking medications.

Results

A 5-item questionnaire was developed to cover the different aspects of medicine taking. 596/1687 (35%) questionnaires were returned. 56% reported getting help in at least one aspect of taking medication and 11% needing more help. 33% reported missing taking their medicines. Unmet needs were associated with receiving help with medications (OR: 5.9, $p<0.001$), being on a higher number of medications (OR: 1.2, $p<0.001$) and being dependent for activities of daily living (ADLs) (OR: 4.9, $p=0.001$). Missing medication was associated with having unmet needs (OR: 5.3, $p<0.001$), receiving help with medications (OR: 2.1, $p<0.001$), being on a higher number of medicines (OR: 1.1, $p=0.008$) and being older than 70 years (OR: 0.6, $p=0.006$).

For peer review only - http://bmjopen.bmj.com/site/about/guidelines.xhtml

Conclusions

More than half of patients who replied needed help with taking medication, and 1 in 10 had unmet needs in this regard. Stroke survivors dependent on others have more unmet needs, more likely to miss medicines and might benefit from focused clinical and research attention. Novel primary care interventions focusing on the practicalities of taking medicines are warranted.

Abstract word count- 298

Keywords: Stroke, Medication Adherence, Caregivers, Barthel

Article summary

Strengths and Limitations

- Development of the questionnaire was based on patients' and caregivers' own views gathered through workshops.
- Stroke survivors were recruited from two UK regions.
- This work identified issues from a population that includes patients severely affected by stroke, who are often excluded from research.
- Results shed light on the effect of stroke related impairments on practical domains and predictors of medicine taking, which have significant effects on medication adherence and call for new primary care interventions.
- The low response rate reported is a limitation of this study and stroke survivors who are harder to reach may have been missed.

Funding: This study was funded by the RCGP SFB, Ref. SFB 2014 – 15 'Quantifying the support stroke survivors get with daily medication taking: a questionnaire survey'. Anna De Simoni and Luis Ayerbe are funded by a NIHR Academic Clinical Lectureships. This article therefore presents independent research funded by NIHR. The views expressed are those of the authors and not necessarily those of the NHS, the NIHR, or the Department of Health. James Jamison was supported by a research grant from The Stroke Association and the British Heart Foundation: TSA BHF 2011/01

For peer review only - http://bmjopen.bmj.com/site/about/guidelines.xhtml

Data sharing statement: No additional data available.

Introduction

Stroke is the leading cause of disability in developed countries, with an estimated that 25-74% of the 50 million stroke survivors worldwide requiring some assistance or being fully dependent on caregivers for activities of daily living (ADLs).[1-3] For many older adults remaining independent at home may depend on how well they can manage complex medication regimens.[4 5] Around half of stroke survivors are dependent on others for everyday activities.[6]

There is evidence that being dependent for ADLs and impairment in mobility and communication decrease medication adherence in patients suffering from hypertension.[7] Deficits in attention, cognition or working memory have been linked with non-adherence to medications in other patient groups.[8] In a recent systematic review of medication adherence among patients with cognitive impairment, one third of studies showed that such patients were likely to have a caregiver to assist with medications and there was an association between taking four or more medicines and nonadherence.[9] In patients taking cardiovascular medicines, multiple factors including cognitive problems, lack of social support, dosing regimen, as well as practical problems and difficulties accessing services, contribute to poor medication adherence.[10 11] Low adherence to secondary prevention medication is associated with poor cardiovascular health.[12 13]

Stroke survivors have previously reported difficulties in the handling of medication as a barrier to adherence to secondary prevention medication after stroke.[14] This was true irrespective of age at stroke, with younger and older stroke survivors being similarly affected.[14] Research on medication adherence in stroke has identified multiple barriers to medication taking among stroke survivors.[14-16] However interventions developed to improve adherence have mainly concentrated on patients responsible for their own medicine taking.[17 18]

In England, the average age at stroke is 74 for men and 80 years for women.[19] In elderly patients in particular, cognitive deficits, taking large number of medicines and the complexity of medication regimens have been identified as barriers to medication adherence. [20 21] Caregivers are known to play a key role in

For peer review only - http://bmjopen.bmj.com/site/about/guidelines.xhtml

providing assistance to older people in a range of daily activities including medication taking and physician visits,[22] and can help improve adherence in cardiac patients with memory problems.[23]

Survivors of stroke have previously reported unmet needs including physical difficulties, cognitive and emotional difficulties, information needs and other unmet needs.[24 25] However we know little about factors that influence medication taking among stroke survivors with disabilities (i.e. physical or cognitive) living in the community (i.e. not in nursing homes), their unmet needs around the use of medicines or the proportion relying on caregivers for some or all aspects of medicine taking.

To date, survey instruments examining the unmet needs of stroke survivors have not focused on practical aspects of medication taking such as how patients collect or handle their medicines.

The aims of this investigation were to design an instrument to evaluate the support stroke survivors get with taking their medicines, characterise patients receiving help with medications, estimate the proportion who have unmet needs with daily medicine taking and who miss medications. We additionally aimed to identify the predictors of missing medicines and of experiencing unmet needs with medications.

This knowledge can inform the development of primary care interventions aimed at improving medication taking in this patients' group.

Methods

Questionnaire development workshops

To develop the questionnaire, current literature evidence was evaluated [17] and three workshops were conducted with 26 stroke survivors and 12 caregivers in the East of England (St John's College, Cambridge 2009 [26]: 7 patients, 1 caregiver; Different Strokes, Cambridge 2012: 9 patients, 3 caregivers; Peterborough, 2012: 10 patients, 8 caregivers). Recruitment was opportunistic and no purposive sampling was applied. The workshops were organised in the context of gathering Patient and Public Involvement (PPI) input into research grant applications aimed at improving adherence to medication after stroke.[17]

The survey questions were developed through thematic analysis [27] of workshops field notes.

For peer review only - http://bmjopen.bmj.com/site/about/guidelines.xhtml

A fourth workshop was conducted to gather feedback on the questionnaire using a PPI (Patient and Public Involvement) exercise with 11 stroke survivors and 3 caregivers recruited through a local stroke group (Different Strokes, East of England). Two stroke survivors from this group took part in subsequent 'think-aloud' interviews, which involved talking out loud as they read the questionnaire, continually verbalising what they were thinking.

Postal survey

In respect to sample size, 400 returned questionnaires would allow good precision for prevalence estimates. The 95% confidence intervals on various proportions with this sample size were calculated using the Wilson score method (with continuity correction) and are as follows: 50% (45.00-55.00%), 25% (20.89%-29.60%), 5% (3.16-7.74%). With 600 questionnaires, the improvement in the precision of the estimates would be as follows: 45.93%-54.07%, 21.62-28.70% and 3.46%-7.14% respectively.

General practices in primary care in the East of England and London were approached through the Clinical Research Network (CRN). CRN Eastern contacted 20 GP practices, of which 11 replied and took part in the study. CRN North London contacted 140 GP East London practices by email (Tower Hamlets, Newham and City & Hackney CCGs), of which only two replied and participated in the study. Five of the eight GP practices contacted in North London (Barnet CCG) through a research coordinator took part in the study.

Patients with stroke and their caregivers were sent the postal questionnaire according to the following criteria

<u>Inclusion Criteria</u>

Patients:

All patients aged > 18 on the practice stroke register with documented history of stroke.

Caregivers:

- Anyone identified by the patient as having a role helping with medicine taking.

<u>Exclusion Criteria</u>

- Patients who suffered a Transient ischaemic attack (TIA) but not a stroke.
- Palliative or end of life patients.

For peer review only - http://bmjopen.bmj.com/site/about/guidelines.xhtml

- Patients receiving institutional long term care (receiving total care in residential homes or living in nursing homes).
- Patients considered unsuitable to taking part in the study by their GP.

Survey participant identification

A list of prospective patients was compiled from the stroke register of each surgery by the practice staff. No restriction was placed on the recruitment of survivors who were dependent for ADLs or lacking capacity. The list was screened by a practice GP and anyone not meeting the inclusion criteria or who was considered unsuitable for the study was excluded. Reasons for unsuitability were not collected for practical reasons.

Survey participant recruitment

Eligible participants were sent a study survey pack by practice staff between September 2016 and February 2017. Study recruitment packs included two invitation letters, information sheets, questionnaires and postal version of Barthel Index[28], one of which was for completion by the patient and the other by the caregiver. The Barthel Index provides a measure of functional independence and physical functioning and has been used in stroke research previously.[29] Patients with Barthel score 20 were categorised as independent for ADLs, those with score 15-19 moderately dependent for ADLs, and those with scores 0-14 severely dependent. [30] If receiving help with medications, the patient was asked to pass to their caregiver the invitation letter and information sheet and invite him/her to complete their copy of the questionnaire, providing answers on the patient's medicine taking. Family members, friends or paid caregivers of stroke survivors who were severely disabled and/or lacked mental capacity were invited to fill and return the caregivers' questionnaires only on behalf of patients. The information sheets stated that consent was implied by returning the completed questionnaire. Participants were asked to return completed questionnaires to the research centre in the FREEPOST envelopes provided. A second mail out of the study invitation pack was sent to all patients as a reminder, 2 weeks after the first one.

For peer review only - http://bmjopen.bmj.com/site/about/guidelines.xhtml

Ethical approval

This study has received ethical approval from Cambridge Central Research Ethics Committee (REC reference: 16/EE/0182) and from the Health Research Authority (IRAS project ID: 170931)

Survey Analysis

Survey data entry was performed by Document Capture Company.[31] Individual patients' characteristics (age, gender, time since stoke, number of daily medicines) were collected from the questionnaires themselves. Practice population, number of patients on stroke registers, deprivation score and ethnicity were taken from the National General Practice profiles (https://fingertips.phe.org.uk/profile/general-practice). The proportions of patients in each sociodemographic category, needing help taking medication, missing any medication in the previous 30 days, and reporting the need for more help taking medication, were estimated. When the survivor and caregiver questionnaires were both returned together, study data were collected from the patient's questionnaire only. The associations between 'Unmet needs' and age (< or ≥70 years), gender, total number of medicines taken, dependence for ADLs, years since stroke, and receiving help with medicines were investigated with individual logistic regression models (a different model per variable investigated), adjusted each and all of them for age and gender. Individual logistic regression models adjusted for age and gender were also used to estimate the association between 'Missed medicines in the previous 30 days' and age (< or > 70 years), gender, total number of medicines taken, dependent for ADLs, years since stroke, help with medicines and unmet needs (a different model adjusted for age and gender per variable investigated). Sensitivity analysis was conducted to investigate if predictors of missing medication or unmet needs vary when the analysis was done on the whole dataset *versus* on questionnaires filled by patients only. Chi squared tests were used to compare the responses on unmet needs and missing medication given by patients *versus* caregivers.

All statistical analysis has been conducted with Stata (version 14, StataCorp LP, College Station, TX, USA, 2013).

For peer review only - http://bmjopen.bmj.com/site/about/guidelines.xhtml

Results

Questionnaire development

Taking medications emerged as an important issue in all three workshops: nearly half of patients stated that a family member or friend was supporting them with daily medicine routines especially in relation to prompting medicine taking. This was put down to effects of the stroke itself on memory retention rather than general memory problems that people without stroke also experience. They admitted missing doses due to forgetting. Only a small proportion of survivors were actually handling their own prescriptions and were relying on support from family and/or community services. In one workshop almost all survivors had Dosette medication boxes and agreed that taking medications out of safety bottles and blister packs was a problem due to physical disabilities.

Thematic analysis of workshop data revealed five main practical domains of support needed with medication taking: 1) Dealing with prescriptions and collection of medicines; 2) Getting medicines out of the box, blister packs of bottles; 3) Prompting 'It's time to take your medicine'; 4) Swallowing medicines; and 5) Checking whether medicines have been taken. The final study questionnaire (see Supplementary file 1) included questions relating to each of these five domains, one item related to adherence (missed medicine in the last 30 days) and an assessment of disabilities through completion of the validated postal version of the Barthel Index.[28] The questionnaire was adapted for caregivers (see Supplementary file 2).

Questionnaire finalisation

On the basis of the fourth workshop and two 'think-aloud' interviews, we reworded the survey questions (e.g. from 'Do you get help with' was changed into 'Is somebody helping you with') and used a scale response 'All the time', 'Often', 'Sometimes', 'Rarely', 'Never' for the first question of each of the five survey domains, which was originally conceived as a 'yes' or 'no' answer (see supplementary file for text of questions).

For peer review only - http://bmjopen.bmj.com/site/about/guidelines.xhtml

Survey

Practice characteristics

18 GP practices agreed to take part in the study, of which just over 1/3 were in London (n=7). GP practices were relatively large with an average population of 11,904 patients (SD = 4010) and a low to moderate level of deprivation (Index of Multiple Deprivation[32] (IMD): Mean-7.05: SD-3.19). Out of 3066 patients on the stroke registers, 1687 stroke patients (55%) were considered eligible for the study and received the postal questionnaire. The average response rate of East of England and London practices was 42% and 27% respectively. The response rate varied between 16% and 53% across practices.

Participant characteristics

596 participants returned a completed questionnaire [549 (92.1 %) from patients, 47 (7.9 %) from caregivers showing a mean response rate of 35% (0.33-0.37). Participants were on average 72.7 yrs old. 37.8% (n=210) of the sample were female, see table 1. There were a high proportion of white patients in the recruited practices (79%), which were on average 21% of mixed or ethnic minority background. Approximately 28% of study participants were completely independent for ADLs.

Participants getting any kind of help with medicines were on average 73.6 years old, two thirds were male with only 19% of this group completely independent for ADLs.

Patients with unmet needs were on average 69 years old, predominantly male (71%) and 56.86% were severely dependent for ADLs. Patients who missed medications were on average 70 years old, 64% were male and the majority (48%) were moderately dependent for ADLs.

Table 1 here

For peer review only - http://bmjopen.bmj.com/site/about/guidelines.xhtml

Support with daily medication taking

Overall, 55.5% (95% CI: 51.7-59.7) of the participants received help in at least one aspect of taking medication, in that they ticked one of the options from 'all the time' to 'rarely' on one or more of the five questions related to medicine taking. 11% (95% CI: 8.8-13.9) of patients reported experiencing unmet needs and needing more help with at least one of the aspects of taking medication, in that they ticked 'yes' to the question "do you feel you need more help", on one or more of the five questions related to medicine taking. The proportion of questionnaires reporting unmet needs filled in by caregivers, 19.6% (n=9), and by patients, 10.7% (n=57), had no significant difference (p=0.068).

Among participants help was needed to some degree with prescriptions and collection of medicines (49.8 %), getting medicines out of the box or packet (27.9 %), reminding to take medicines (36.4 %), swallowing medicines (20.2 %) and checking that medicines have been taken (34.1 %).(see Table 2). Being reminded to take medicines, dealing with prescriptions and collection of medicines and getting medicines out of a pack or bottle were the most commonly reported areas of unmet needs. Almost two thirds of participants (65.3%) reported never missing medicines in the last 30 days. Out of the 34.7% of patients who said they missed taking medicine at any point in the previous 30 days, 23.9% said rarely, 9.3% sometimes, 0.8% often and 0.7% all the time. The proportion of questionnaires reporting missing medication at some point, filled in by caregivers, 27.7% (n=13), and by patients 35.3% (n=193), had no significant difference (p=0.292).

Table 2 here

Factors associated with unmet needs

Being on a higher total number of daily medications (OR: 1.2, (1.1-1.3), p<0.001), severe dependence for ADLs (OR: 11.6 (4.2-32.4) p<0.001) and receiving any kind of help (OR: 5.9, (2.7-11.6), p<0.001) in relation to taking medication was associated with experiencing unmet needs. Getting help with swallowing medicines (OR: 6.8, (3.8-12.0), p<0.001), getting medicines out of a box, blister packs or bottles (OR: 6.6, (3.6-11.8), p<0.001) showed the strongest associations with experiencing unmet needs (see table 3).

For peer review only - http://bmjopen.bmj.com/site/about/guidelines.xhtml

When the analyses were conducted with data from questionnaires filled by patients only, the variables significantly associated with unmet needs were the same, apart from years since stroke (Supplementary Appendix 1).

Table 3 here

Factors associated with missing medications

Being older (age ≥70) was associated with a lower probability of missing medication (OR: 0.6 (0.4-0.9) p=0.006). Being on a higher number of daily medicines (polypharmacy) (OR: 1.1 (1.0-1.1), p=0.008) and getting any kind of help with medicine taking (OR:2.1 (1.4-3.0) p<0.001) was associated with higher probability of missing medicines. The more unmet needs stroke survivors had with taking medication, the more likely they were to miss their medicines (OR: 5.3 (3.0-9.4), p<0.001). (see Table 4). When the analyses were conducted with data from questionnaires filled by patients only, the variables significantly associated with missing medication were the same (Supplementary Appendix 1).

Table 4 here

Discussion

Summary of findings

From workshops we identified 5 key issues that patients regarded as important with medication taking after stroke. We converted these into a five item questionnaire that we distributed to people on stroke registers in 18 general practices. We obtained a response rate of 35%. Among respondents, 56% of survivors in the community were receiving help in some aspect of daily medication taking, 11% reported needing more help in

For peer review only - http://bmjopen.bmj.com/site/about/guidelines.xhtml

For peer review only

at least one domain of medicine taking and 34% missed taking their medicines at some point in the previous 30 days.

A higher total number of daily medicines, being severely dependent for ADLs and receiving help with medication were predictors of experiencing at least one unmet need in respect of medication taking. Stroke survivors who were younger, taking a higher number of daily medicines and experiencing a greater number of unmet needs were more likely to miss medications.

This work identified issues from a population that includes patients severely affected by stroke, who are often excluded from research.[17] Results presented here shed light on the effect of stroke related impairments on practical domains and predictors of medicine taking, which are shown to have significant effects on overall adherence.

Strengths and limitations

A strength of this study is that the questionnaire was developed from patients' and caregivers' own views gathered through workshops. Although not recruited through purposive sampling, workshop participants suffered from a range of stroke related impairments, as highlighted by the reported use of Dossette boxes, dependence on others for aspects of medicine taking like prompting medication times, and dependence for ADLs such as collecting prescriptions and taking tablets out of boxes. In the postal survey, the inclusion of stroke survivors regardless of level of dependence for ADLs permitted investigating a population who are understudied,[17] yet may have significant unmet needs that can affect their adherence to medications. This investigation highlights caregivers' role in managing medicines in survivors dependent for ADLs.

However, study limitations should also be considered. The response rate across recruited GP practices was low and harder to reach stroke survivors may have been missed. Poor response rate is a source of bias that might affect our estimates.

Interestingly, considering the average age at stroke in England (i.e. 74 for men and 80 years for women[19]), our participants' population was slightly younger (73 years), perhaps reflecting the fact that patients receiving institutional long term care were excluded from the study or that older people found harder taking part in a postal survey. Through the Barthel score, we did not assess cognition directly, although low cognitive function

For peer review only - http://bmjopen.bmj.com/site/about/guidelines.xhtml

is associated with poor adherence.[33] As the Barthel focuses on physical disability it is not known to what extent study participants were cognitively impaired or suffered from communication difficulties like aphasia. In addition, dependency for ADLs could have been caused by existing co-morbidities other than stroke. We did not collect information on the use of blister packaged medication or devices to aid compliance, which could have influenced medication taking practices. Finally this study examined all medicine taking and did not differentiate between stroke secondary prevention medications and other drug categories.

Comparisons with existing research

To our knowledge this is the first study that shows that more than half of all stroke survivors get help with some aspect of medicine taking and that those receiving help are more likely to have unmet needs. This provides some insight in to why adherence to medication in stroke survivors may be poor.[34]

Moreover, the greater the number of medicines, the more likely stroke survivors were to miss medications. Addressing pill burden by simplifying drug regimens may be an important focus for future interventions. Indeed the polypill approach to medication taking has been shown to reduce cardiovascular as well as total pill burden in a primary care setting.[35] Simpler dosing regimens, are known to be associated with better medication adherence [36] while fewer medicines has been shown to be an independent predictor of long term medication persistence among stroke survivors.[37 38] A recent trial incorporating a fixed-dose combination polypill approach to taking cardiovascular medicine demonstrated better adherence among patients receiving a single pill.[39]

Receiving help with prescriptions and collecting medicines was identified as the area where most help was received (49.7% of respondents). Stroke survivors who are dependent for activities of daily living may face considerable practical challenges accessing health care resources at the pharmacy and the GP practice. A recent study in the USA found that around 2/3 of caregivers were involved in at least 1 medication management activity of elderly patients and that high involvement in Instrumental Activities of Daily Living (IADLs) was associated with the caregiver providing the patient with assistance in ordering medicines.[40] Filling prescriptions is also known to be an important factor influencing medication adherence.[41 42] Indeed caregivers can play a significant role in ensuring appropriate medication taking. A recent interview study exploring potential barriers and facilitators of medication adherence in stroke identified the central role of the

For peer review only - http://bmjopen.bmj.com/site/about/guidelines.xhtml

caregiver in medication adherence.[43] Our evaluation of an online stroke forum also confirmed the important role of the caregiver in facilitating medication adherence.[14] Monitoring prescription collections, liaising with the GP and pharmacy, increasing the time between prescriptions or arranging medication deliveries, may help to address prescription needs.

Around 11% of stroke survivors reported unmet medication needs. We found that stroke survivors severely dependent for ADLs and receiving help with medicines were more likely to report unmet needs, which is in line with a recent study investigating stroke/TIA survivors in Australia, where greater functional ability was associated with fewer unmet needs, including those related to secondary prevention.[44] In previous research on unmet needs among stroke survivors, a 44 item survey study by McKevitt and colleagues (2011) reported that 49% of stroke survivors had at least one unmet need [25], while in a study of Australian survivors who completed a 58 item survey, the percentage was 84%.[24] Both these studies however examined unmet needs over a variety of domains including health, work, leisure and everyday living, social support and finances, whereas our study focused on medication needs only.

Getting help to take medicines out of a box, packet or bottle was the area where the greatest proportion of stroke survivors needed help all of the time. We previously found that the use of pill boxes and blister packed medication to be both a facilitator [35] and a barrier [14] to adherence among stroke survivors[15], while interventions using blister packaging and pill boxes have been found to be associated with improved adherence.[45] Although electronic medication devices were considered potentially effective in improving medication taking behaviour among patients with cognitive impairments, success in using such devices was dependent on the patient having a good level of dexterity, while removing the medication from these devices was also found to be challenging. [46 47 48]

The need for further support in this domain, as reported in the current study, suggests that handling medications remains problematic for stroke survivors.

An interesting finding from this survey study is that stroke survivors who missed medicines were younger. This is consistent with other research on adherence in stroke that found that younger age was predictive of poor adherence[49], and has also been described in patients taking medication for cardiovascular disease.[50] The finding in the present study contrasts with the view that older patients are more likely to face difficulties taking medication[51 52] which is frequently attributed to higher number of pre-existing comorbidities

For peer review only - http://bmjopen.bmj.com/site/about/guidelines.xhtml

resulting in polypharmacy and increased complexity of medication taking regimens. The fact that older patients may be less likely miss medicine might be down to the support they receive from caregivers. Our findings suggests that support needed with medications may be overlooked in younger stroke survivors.[53]

In this study a significant proportion of patients admitted missing medications occasionally. There is evidence that improving adherence by one anti-hypertensive pill/week for a once-a-day regimen reduces the hazard of stroke by 8–9 % and death by 7 %.[54] Each incremental 25% increase in proportion of days covered with statin medications is associated with a 0.10 mmol/L reduction in LDL-C cholesterol.[55 56] Non-adherence to cardiovascular medications is associated with increased risk of morbidity and mortality.[57]

Implications for clinical practice

A significant proportion of patients, particularly those who take large numbers of tablets, are disabled or receive help to take medication, have unmet needs and miss their tablets, which can increase risk of recurrent cardiovascular events. These particularly vulnerable groups of patients might benefit from focused clinical attention. Through understanding the needs of survivors and caregivers in different aspects of daily medication taking, we can help direct future resources to the areas of greatest need. For example, further exploration of medication packaging is warranted to understand the difficulties stroke survivors face handling medicines. Polypharmacy remains a difficulty for older patients. Therefore, exploring the use of combination pills and further efforts to reduce the burden of multiple medications among stroke survivors is warranted.

The questionnaire we have developed could be used to understand the challenges around medication faced by other patient groups. Unmet medication needs among UK stroke survivors have not been previously explored in the context of activities both survivors and caregivers consider important for taking medicines. Through understanding the extent of unmet needs as well as the areas in which these are greatest, strategies can be developed which address poor medication taking practices and therefore improve medication adherence.

For peer review only - http://bmjopen.bmj.com/site/about/guidelines.xhtml

Future research

Novel interventions focussing on the practicalities of taking medicines and aimed at improving stroke survivors' adherence to treatment are needed. The findings reported here may inform the development of such interventions. Advances in technology have the potential to facilitate delivery of such interventions, e.g. electronic devices prompting medication taking times.[58 59] Efforts to improve medication taking among survivors of stroke using technology are already underway and have shown promise.[60].

Acknowledgements

The authors wish to thank all the stroke survivors and caregivers who participated in this study.

Competing interests

None declared

Data sharing statement

No additional data are available

Author/s contribution

ADS is the Chief Investigator, contributed to the study design, data analysis and commented on the manuscript. JJ contributed to the study design, data collection, data analysis and prepared the manuscript for submission. JM is a co-investigator on the study, wrote and commented on the manuscript. SS is a co-investigator on the study, wrote and commented on the manuscript. LA contributed to the data analysis and commented on the manuscript. GDiT contributed to the data analysis and commented on the manuscript. All authors agreed on the final draft of the submitted manuscript.

For peer review only

References

1. Young J, Forster A. Review of stroke rehabilitation. *BMJ* 2007;334(7584):86-90. doi: 10.1136/bmj.39059.456794.68

2. Miller EL, Murray L, Richards L, et al. Comprehensive overview of nursing and interdisciplinary rehabilitation care of the stroke patient: a scientific statement from the American Heart Association. *Stroke* 2010 (Oct);41(10):2402-48. doi: 10.1161/STR.0b013e3181e7512b

3. Feigin VL, Barker-Collo S, Parag V, et al. Auckland Stroke Outcomes Study: Part 1: Gender, stroke types, ethnicity, and functional outcomes 5 years poststroke. *Neurology* 2010;75(18):1597-607. doi: 10.1212/WNL.0b013e3181fb44b3

4. Marek KD AL. Medication Management of the Community-Dwelling Older Adult. In: RG H, ed. An Evidence-Based Handbook for Nurses: Agency for Healthcare Research and Quality (US). Rockville, MD2008.

5. Lewis A. Non-compliance: A $100 billion problem. . *The Remington Report* 1997;5:14-5.

6. National Audit Office. Progress in improving stroke care. In: Office NA, ed. London: Department of Health, 2010.

7. Park JH, Park JH, Lee SY, et al. Disparities in Antihypertensive Medication Adherence in Persons With Disabilities and Without Disabilities: Results of a Korean Population-Based Study. *Archives of Physical Medicine and Rehabilitation* 2008;89(8):1460-67. doi: http://dx.doi.org/10.1016/j.apmr.2007.12.045

8. Stilley CS, Bender CM, Dunbar-Jacob J, et al. The Impact of Cognitive Function on Medication Management: Three Studies. *Health psychology : official journal of the Division of Health Psychology, American Psychological Association* 2010;29(1):50-55. doi: 10.1037/a0016940

9. Smith D, Lovell J, Weller C, et al. A systematic review of medication non-adherence in persons with dementia or cognitive impairment. *PLoS ONE* 2017;12(2):e0170651. doi: 10.1371/journal.pone.0170651

10. Kronish IM, Ye S. Adherence to Cardiovascular Medications: Lessons Learned and Future Directions. *Progress in cardiovascular diseases* 2013;55(6):590-600. doi: 10.1016/j.pcad.2013.02.001

For peer review only

11. Gordon K, Smith F, Dhillon S. Effective chronic disease management: Patients' perspectives on medication-related problems. *Patient Education and Counseling* 2007;65(3):407-15. doi: http://dx.doi.org/10.1016/j.pec.2006.09.012

12. Xu J, Zhao X, Wang Y, et al. Impact of a Better Persistence with Antihypertensive Agents on Ischemic Stroke Outcomes for Secondary Prevention. *PLoS ONE* 2013;8(6):e65233. doi: 10.1371/journal.pone.0065233

13. Colivicchi F, Bassi A, Santini M, et al. Discontinuation of Statin Therapy and Clinical Outcome After Ischemic Stroke. *Stroke* 2007;38(10):2652-57. doi: 10.1161/strokeaha.107.487017

14. Jamison J, Sutton S, Mant J, et al. Barriers and facilitators to adherence to secondary stroke prevention medications after stroke: Analysis of survivors and caregivers views from an online stroke forum. *BMJ Open* 2017;7 (e016814)

15. Jamison J, Graffy J, Mullis R, et al. Barriers to medication adherence for the secondary prevention of stroke: a qualitative interview study in primary care. *The British Journal of General Practice* 2016;66(649):e568-e76. doi: 10.3399/bjgp16X685609

16. Souter C, Kinnear A, Kinnear M, et al. Optimisation of secondary prevention of stroke: a qualitative study of stroke patients' beliefs, concerns and difficulties with their medicines. *International Journal of Pharmacy Practice* 2014;22(6):424-32. doi: 10.1111/ijpp.12104

17. De Simoni A, Mant J, Sutton S. Adherence to medication in stroke survivors dependent on caregivers. *British Journal of General Practice* 2015;65(640):e789.

18. De Simoni A, Hardeman W, Mant J, et al. Trials to Improve Blood Pressure Through Adherence to Antihypertensives in Stroke/TIA: Systematic Review and Meta-Analysis. *Journal of the American Heart Association* 2013 (Aug 20);2(4):e000251. doi: 10.1161/jaha.113.000251

19. Royal College of Physicians. Sentinel Stroke National Audit Programme (SSNAP). National clinical audit annual results portfolio March 2015-April 2016. [Available from: http://bit.ly/1M5R3Op

20. Munger MA, Van Tassell BW, LaFleur J. Medication Nonadherence: An Unrecognized Cardiovascular Risk Factor. *Medscape General Medicine* 2007;9(3):58-58.

21. Brown MT, Bussell JK. Medication Adherence: WHO Cares? *Mayo Clinic proceedings* 2011;86(4):304-14. doi: 10.4065/mcp.2010.0575

For peer review only - http://bmjopen.bmj.com/site/about/guidelines.xhtml

22. Wolff JL, Spillman B. Older Adults Receiving Assistance With Physician Visits and Prescribed Medications and Their Family Caregivers: Prevalence, Characteristics, and Hours of Care. *The Journals of Gerontology: Series B* 2014;69(Suppl_1):S65-S72. doi: 10.1093/geronb/gbu119

23. Ownby RL, Hertzog C, Crocco E, et al. Factors related to medication adherence in memory disorder clinic patients. *Aging & mental health* 2006;10(4):378-85. doi: 10.1080/13607860500410011

24. Andrew NE, Kilkenny M, Naylor R, et al. Understanding Long-Term Unmet Needs in Australian Survivors of Stroke. *International Journal of Stroke* 2014;9(SA100):106-12. doi: doi:10.1111/ijs.12325

25. McKevitt C, Fudge N, Redfern J, et al. Self-Reported Long-Term Needs After Stroke. *Stroke* 2011;42(5):1398-403. doi: 10.1161/strokeaha.110.598839

26. De Simoni A, Kellar I, Sutton S, et al. Developing a pharmacist intervention to improve adherence to hypertensive medications in TIA/stroke patients. SAPC Conference. Norwich, 2010.

27. Braun V, Clarke V. Using thematic analysis in psychology. *Qualitative Research in Psychology* 2006;3(2):77-101. doi: 10.1191/1478088706qp063oa

28. Gompertz P, Pound P, Ebrahim S. A postal version of the Barthel Index. *Clinical Rehabilitation* 1994;8(3):233-39. doi: doi:10.1177/026921559400800308

29. Quinn TJ, Langhorne P, Stott DJ. Barthel Index for Stroke Trials. *Development, Properties, and Application* 2011;42(4):1146-51. doi: 10.1161/strokeaha.110.598540

30. Wolfe CD, Taub NA, Woodrow EJ, et al. Assessment of scales of disability and handicap for stroke patients. *Stroke* 1991;22(10):1242-44. doi: 10.1161/01.str.22.10.1242

31. Document Capture Company [Available from: http://www.documentcapture.co.uk/data-collection-methods/

32. Smith T, Noble, M, Noble S, et al. The English Indices of Deprivation 2015 Research report, 2015.

33. Hayes TL, Larimer N, Adami A, et al. Medication Adherence in Healthy Elders. *Journal of Aging and Health* 2009;21(4):567-80. doi: 10.1177/0898264309332836

34. Chowdhury R, Khan H, Heydon E, et al. Adherence to cardiovascular therapy: a meta-analysis of prevalence and clinical consequences. *European Heart Journal* 2013;34:2940-8. doi: 10.1093/eurheartj/eht295

For peer review only - http://bmjopen.bmj.com/site/about/guidelines.xhtml

35. Truelove M, Patel A, Bompoint S, et al. The Effect of a Cardiovascular Polypill Strategy on Pill Burden. *Cardiovascular Therapeutics* 2015;33(6):347-52. doi: 10.1111/1755-5922.12151

36. Claxton AJ, Cramer, J., Pierce, C. A Systematic Review of the Associations Between Dose Regimens and Medication Compliance *Clinical therapeutics* 2001;23(8):1296-310.

37. Bushnell CD, Olson DM, Zhao X, et al. Secondary preventive medication persistence and adherence 1 year after stroke. *Neurology* 2011;77(12):1182-90. doi: 10.1212/WNL.0b013e31822f0423 [published Online First: 2011/09/09]

38. Chapman RH, Benner JS, Petrilla AA, et al. Predictors of adherence with antihypertensive and lipid-lowering therapy. *Archives of Internal Medicine* 2005;165(10):1147-52. doi: 10.1001/archinte.165.10.1147

39. Thom S, Poulter N, Field J, et al. Effects of a fixed-dose combination strategy on adherence and risk factors in patients with or at high risk of CVD: the UMPIRE randomized clinical trial. *JAMA : the journal of the American Medical Association* 2013;310(9):918-29. doi: 10.1001/jama.2013.277064

40. Noureldin M, Plake KS. Correlates of caregivers' involvement in the management of older adults' medications. *Research in Social and Administrative Pharmacy* 2017;13(4):840-48. doi: https://doi.org/10.1016/j.sapharm.2016.09.009

41. Andersson K, Melander A, Svensson C, et al. Repeat prescriptions: refill adherence in relation to patient and prescriber characteristics, reimbursement level and type of medication. *European Journal of Public Health* 2005;15(6):621-26. doi: 10.1093/eurpub/cki053

42. Vlasnik JJ, Aliotta SL, DeLor B. Medication adherence: Factors influencing compliance with prescribed medication plans. *The Case Manager* 2005;16(2):47-51. doi: http://dx.doi.org/10.1016/j.casemgr.2005.01.009

43. Bauler S, Jacquin-Courtois S, Haesebaert J, et al. Barriers and Facilitators for Medication Adherence in Stroke Patients: A Qualitative Study Conducted in French Neurological Rehabilitation Units. *European Neurology* 2014;72(5-6):262-70.

44. Olaiya MT, Cadilhac DA, Kim J, et al. Long-term unmet needs and associated factors in stroke or TIA survivors: An observational study. *Neurology* 2017 (Jul 4);89(1):68-75. doi: 10.1212/wnl.0000000000004063

For peer review only - http://bmjopen.bmj.com/site/about/guidelines.xhtml

45. Conn VS, Ruppar TM, Chan KC, et al. Packaging interventions to increase medication adherence: systematic review and meta-analysis. *Current Medical Research and Opinion* 2015;31(1):145-60. doi: 10.1185/03007995.2014.978939

46. Hall J, Bond C, Kinnear M, et al. Views of patients and professionals about electronic multicompartment medication devices: a qualitative study. *BMJ Open* 2016;6:e012915. doi: 10.1136/bmjopen-2016-012915

47. Beckman AGK, Parker MG, Thorslund M. Can elderly people take their medicine? *Patient Education and Counseling* 2005;59(2):186-91. doi: http://dx.doi.org/10.1016/j.pec.2004.11.005

48. Notenboom K, Beers E, van Riet-Nales DA, et al. Practical Problems with Medication Use that Older People Experience: A Qualitative Study. *Journal of the American Geriatrics Society* 2014;62(12):2339-44. doi: 10.1111/jgs.13126

49. O'Carroll R, Whittaker J, Hamilton B, et al. Predictors of adherence to secondary preventive medication in stroke patients. *Ann Behav Med* 2011;41(3):383-90. doi: 10.1007/s12160-010-9257-6 [published Online First: 2011/01/05]

50. Crowley MJ, Zullig LL, Shah BR, et al. Medication Non-Adherence After Myocardial Infarction: An Exploration of Modifying Factors. *Journal of General Internal Medicine* 2015;30(1):83-90. doi: 10.1007/s11606-014-3072-x

51. Hughes CM. Medication Non-Adherence in the Elderly. *Drugs & Aging* 2004;21(12):793-811. doi: 10.2165/00002512-200421120-00004

52. De Schryver EL, van Gijn J, Kappelle LJ, et al. Non-adherence to aspirin or oral anticoagulants in secondary prevention after ischaemic stroke. *J Neurol* 2005;252(11):1316-21. doi: 10.1007/s00415-005-0858-0 [published Online First: 2005/05/04]

53. Kersten P, Low JTS, Ashburn A, et al. The unmet needs of young people who have had a stroke: results of a national UK survey. *Disability and Rehabilitation* 2002;24(16):860-66. doi: 10.1080/09638280210142167

54. Bailey JE, Wan JY, Tang J, et al. Antihypertensive medication adherence, ambulatory visits, and risk of stroke and death. *J Gen Intern Med* 2010;25(6):495-503. doi: 10.1007/s11606-009-1240-1 [published Online First: 2010/02/19]

For peer review only - http://bmjopen.bmj.com/site/about/guidelines.xhtml

55. Amarenco P, Labreuche J. Lipid management in the prevention of stroke: review and updated meta-analysis of statins for stroke prevention. *The Lancet Neurology* 2009;8(5):453-63. doi: 10.1016/s1474-4422(09)70058-4 [published Online First: 2009/04/21]

56. Ho PM, Rumsfeld JS, Masoudi FA, et al. Effect of medication nonadherence on hospitalization and mortality among patients with diabetes mellitus. *Arch Intern Med* 2006;166(17):1836-41. doi: 10.1001/archinte.166.17.1836 [published Online First: 2006/09/27]

57. Rasmussen JN, Chong A, Alter DA. Relationship between adherence to evidence-based pharmacotherapy and long-term mortality after acute myocardial infarction. *Jama* 2007;297(2):177-86. doi: 10.1001/jama.297.2.177 [published Online First: 2007/01/11]

58. Tao D, Xie L, Wang T, et al. A meta-analysis of the use of electronic reminders for patient adherence to medication in chronic disease care. *Journal of Telemedicine and Telecare* 2015;21(1):3-13. doi: doi:10.1177/1357633X14541041

59. Thakkar J, Kurup R, Laba T, et al. Mobile telephone text messaging for medication adherence in chronic disease: A meta-analysis. *JAMA Internal Medicine* 2016;176(3):340-49. doi: 10.1001/jamainternmed.2015.7667

60. Kamal AK, Shaikh Q, Pasha O, et al. A randomized controlled behavioral intervention trial to improve medication adherence in adult stroke patients with prescription tailored Short Messaging Service (SMS)-SMS4Stroke study. *BMC Neurology* 2015;15(1):212. doi: 10.1186/s12883-015-0471-5

For peer review only

	All patients				Patients who receive any kind of help				Patients with unmet needs				Patients who miss medication			
	N	%	Mean	SD	N	%	Mean	SD	N	%	Mean	SD	N	%	Mean	SD
Age	588		72.7	11.6	331		73.6	12.2	64		68.8		203		70.5	13.0
Female	210	37.8			112	36.2			18	28.6			68	35.6		
Male	346	67.2			197	63.2			45	71.4			123	64.4		
Time since stroke	535		7.7	7.6	295		7.97	8.5	61		9.3	9.2	186		7.7	8.5
N of daily medicines	557		6.4	4	312		7.3	4.5	59		9.7	7.1	190		6.9	4.1
Independent for ADLs (BI=20)	139	28.3			53	18.9			5	9.8			45	25.7		
Moderately dependent for ADLs (BI=15-19)	231	47.1			130	46.4			17	33.3			84	48.0		
Severely dependent for ADLs (BI=0-14)	121	24.6			97	34.6			29	56.9			46	26.3		

Table 1. Characteristics of participants who took part in the survey study *(mean scores reported unless otherwise stated).* N represents the number of participants who completed the survey in respect to the different variables. BI: Barthel Index.

For peer review only

For peer review only - http://bmjopen.bmj.com/site/about/guidelines.xhtml

	N	All the Time N (%)	Often N (%)	Sometimes N (%)	Rarely N (%)	Never N (%)	Yes N (%)	No N (%)
Question 1<br>Is somebody helping with prescriptions and collection of your medicines?	583	186 *(31.9)*	19 *(3.3)*	40 *(6.9)*	45 *(7.7)*	293 *(50.2)*		
Question 1a<br>Do you feel you need more help with prescriptions and collection of your medicines?	551						33 *(6.0)*	518 *(94.0)*
Question 2<br>Is somebody helping you getting the medicines out of the box, bottle or blister pack?	578	85 *(14.7)*	15 *(2.6)*	31 *(5.4)*	30 *(5.2)*	417 *(72.1)*		
Question 2a<br>Do you feel you need more help with getting the medicines out of the box, bottle or blister pack?	553						33 *(6.0)*	520 *(94.0)*
Question 3<br>Is somebody helping with reminding you when is the time to take your medicine?	577	78 *(13.6)*	22 *(3.8)*	59 *(10.2)*	51 *(8.8)*	367 *(63.6)*		
Question 3a<br>Do you feel you need more help with reminding when is the time to take your medicine?	564						35 *(6.2)*	529 *(93.8)*
Question4<br>Is somebody helping you with swallowing your medicine?	579	56 *(9.7)*	11 *(1.9)*	29 *(5.0)*	21 *(3.6)*	462 *(79.8)*		
Question 4a<br>Do you feel you need more help with swallowing your medicine?	560						9 *(1.6)*	551 *(98.4)*
Question 5<br>I somebody helping you with checking that you have taken your medicines	576	76 *(13.2)*	23 *(4.0)*	58 *(10.0)*	40 *(6.9)*	379 *(65.9)*		
Question 5 a<br>Do you feel you need more help with checking that you have taken your medicine	558						20 *(3.6)*	538 *(96.4)*
Thinking of the last 30 days, how often did you miss taking your regular medicines?	594	4 *(0.7)*	5 *(0.8)*	55 *(9.3)*	142 *(23.9)*	388 *(65.3)*		

Table 2. Results summarising participants' responses to the survey questions.

For peer review only - http://bmjopen.bmj.com/site/about/guidelines.xhtml

	Univariable analysis		Multivariable analysis	
**Variable**	**N**	**Odds ratio (95% CI) p value**	**N**	**Odds ratio (95% CI) p value**
Age ≥70	581	0.6 (0.4-1.1) p=0.084	544	0.7 (0.4-1.2) p=0.180
Gender (female)	544	0.7 (0.4-1.2) p=0.137	544	0.7 (0.4-1.2) p=0.147
Number of different medicines	542	1.2 (1.1-1.3) p<0.001	509	1.2 (1.1-1.3) p<0.001
Moderate Dependence for ADLs (BI: 15-19)	479	2.2 (0.8-6.1) p=0.135	447	2.7 (1.0-7.5) p=0.068
Severe Dependence for ADLs (BI: 0-14)	479	8.5 (3.2-22.8) p<0.001	447	11.6 (4.2-32.4) p<0.001
Years since stroke	522	1.0 (1.0-1.1) p=0.078	490	1.0 (1.0-1.1) p=0.160
Getting help with prescriptions and collection of medication	568	4.7 (2.5-8.8) p<0.001	533	4.6 (2.4-8.7) p<0.001
Getting help with taking medicines out of the box, bottle or blister pack	563	6.7 (3.8-11.8) p<0.001	527	6.6 (3.6-11.8) p<0.001
Getting help with reminding you when is the time to take your medicine?	562	4.7 (2.7-8.2) p<0.001	526	4.3 (2.4-7.6) p<0.001
Getting help to swallow the medication	565	6.7 (3.9-11.6) p<0.001	528	6.8 (3.8-12.0) p<0.001
Getting help by checking that you have taken your medicines	562	4.9 (2.8-8.6) p<0.001	526	5.9 (3.1-10.1) p<0.001
Getting any kind of help	574	5.9 (2.8-12.1) p<0.001	537	5.9 (2.7-11.6) p<0.001

Table 3. Results of the multivariable analysis showing the variables associated with unmet needs.
N: number of observations; ADLs: Activities of daily living; BI: Barthel Index.

For peer review only

For peer review only - http://bmjopen.bmj.com/site/about/guidelines.xhtml

	Univariable analysis		Multivariable analysis	
**Variable**	**N**	**Odds Ratio (95% CI) p value**	**N**	**Odds Ratio (95% CI) p value**
Age ≥70	594	0.6 (0.4-0.8) p=0.003	555	0.6 (0.4-0.9) p=0.006
Gender (female)	555	0.9 (0.6-1.2) p=0.401	555	0.9 (0.6-1.3) p=0.498
Number of different medicines	555	1.0 (1.0-1.1) p=0.040	520	1.1 (1.0-1.1) p=0.008
Moderate Dependence for ADLs (BI: 15-19)	490	1.2 (0.8-1.8) p=0.468	456	1.3 (0.8-2.0) p=0.343
Severe dependence for ADLs (BI 0-14)	490	1.3 (0.8-2.1) p=0.342	456	1.4 (0.8-2.4) p=0.239
Years since stroke	533	1.0 (0.9-1.0 p=0.950	499	1.0 (0.9-1.0) p=0.971
Getting help with prescriptions and collection of medication	581	2.0 (1.5-2.9) p<0.001	544	2.3 (1.6-3.3) p<0.001
Getting help to have the medicines out of the box, bottle or blister pack	576	1.4 (1.0-2.0) p=0.089	538	1.5 (1.0-2.2) p=0.051
Getting help with reminding you when is the time to take your medicine?	575	2.5 (1.7-3.6) p<0.001	537	2.7 (1.8-3.9) p<0.001
Getting help to swallow the medication	578	1.5 (1.0-2.3) p=0.045	539	1.7 (1.1-2.6) p=0.022
Getting help by checking that you have taken your medicines	576	2.4 (1.7-3.4) p<0.001	537	2.5 (1.7-3.7) p<0.001
Getting any kind of help	587	2.1 (1.4-3.0) p<0.001	548	2.1 (1.4-3.0) p<0.001
Unmet needs (participant reported more help needed)	580	5.3 (3.0-9.2) p<0.000	544	5.3 (3.0-9.4) p<0.001

Table 4. Results of univariable and multivariable analysis showing associations with missing medicines.
N: number of observations; ADLs: Activities of daily living; BI: Barthel Index.

For peer review only

For peer review only - http://bmjopen.bmj.com/site/about/guidelines.xhtml

	Unmet needs		Missing medication	
**Variable**	**N**	**Odds ratio (95% CI) p value**	**N**	**Odds ratio (95%CI) p value**
Age ≥70	498	0.7 (0.4-1.2) p=0.248	508	0.6 (0.4-0.9) p=0.009
Gender (female)	498	0.7 (0.4-1.3) p=0.262	508	0.9 (0.6-1.3) p=0.576
Number of different medicines	465	1.2 (1.1-1.3) p<0.001	475	1.1 (1.0-1.1) p=0.015
Moderate Dependence for ADLs (BI: 15-19)	408	2.6 (0.9-7.5) p<0.075	417	0.3 (-0.2-0.7) p=0.289
Severe Dependence for ADLs (BI: 0-14)	408	10.9 (3.8-31.0) p<0.001	417	0.5 (-0.1-1.0) p=0.119
Years since stroke	446	1.0 (1.0-1.1) p=0.036	454	1.0 (0.9-1.03) p=0.725
Getting help with prescriptions and collection of medication	487	4.6 (2.4-8.9) p<0.001	497	2.6 (1.8-3.8) p<0.001
Getting help with taking medicines out of the box, bottle or blister pack	481	6.6 (3.6-12.2) p<0.001	491	1.7 (1.1-2.6) p=0.20
Getting help with reminding you when is the time to take your medicine?	480	4.7 (2.6-8.5) p<0.001	490	3.0 (2.0-4.5) p<0.001
Getting help to swallow the medication	482	7.8 (4.2-14.8) p<0.001	492	2.1 (1.3-3.4) p=0.005
Getting help by checking that you have taken your medicines	480	5.9 (3.2-10.9) p<0.001	490	2.8 (1.8-4.2) p<0.001
Getting any kind of help	491	5.6 (2.7-11.9) p<0.001	501	2.3 (1.6-3.4) p<0.001

Supplementary Appendix 1. Predictors of unmet needs and missing medication, responses given by patients only (i.e. caregivers' filled questionnaire excluded). Multivariable analyses, all models adjusted for age and gender. N: number of observations.

For peer review only

Queen Mary University of London

UNIVERSITY OF CAMBRIDGE

QUESTIONNAIRE – PATIENT

Many people with stroke need help with taking their medicines. We would like to ask you few questions about the help you might need with taking your medicines.

How many different type of medicines do you take in one day? ☐
Write the number or an approximation. Please count all medications including eye drops, injections etc.

How old are you? ☐ How long ago was your stroke? ☐ years

What is your sex? M ☐ F ☐

For each question below, please tick the box that best describes how you have taken your medicines in the last month:

1. **Is somebody helping with prescriptions and collection of your medicines?**

   all the time ☐
   often ☐
   sometimes ☐
   rarely ☐
   never ☐

   Do you feel you need more help with prescriptions and collection of your medicines?

   Yes ☐ **No** ☐

2. **Is somebody helping you getting the medicines out of the box, bottle or blister pack?**

   all the time ☐
   often ☐
   sometimes ☐
   rarely ☐
   never ☐

   Do you feel you need more help with getting the medicines out of the box, bottle or blister pack?

   Yes ☐ **No** ☐

For peer review only

3. **Is somebody helping with reminding you when is the time to take your medicine?**

all the time ☐
often ☐
sometimes ☐
rarely ☐
never ☐

Do you feel you need more help with reminding when is the time to take your medicine?

Yes ☐ **No** ☐

4. **Is somebody helping you with swallowing your medicine? For example by giving you a drink**

all the time ☐
often ☐
sometimes ☐
rarely ☐
never ☐

Do you feel you need more help with swallowing your medicine?

Yes ☐ **No** ☐

5. **Is somebody helping you with checking that you have taken your medicines?**

all the time ☐
often ☐
sometimes ☐
rarely ☐
never ☐

Do you feel you need more help with checking that you have taken your medicine?

Yes ☐ **No** ☐

Missing medicines

Thinking of the last 30 days, how often did you miss taking your regular medicines?

Remember - tick one box only

all the time ☐
often ☐
sometimes ☐
rarely ☐
never ☐

For peer review only

Barthel Questionnaire

These are some questions about your ability to look after yourself.

They may not seem to apply to you.

Please answer them all.

Tick one box in each section.

Bathing

In the bath or shower do you:

Remember - tick one box only

- manage on your own? ☐
- need help getting in and out? ☐
- need other help? ☐
- never have a bath or shower? ☐
- need to be washed in bed? ☐

Stairs

Do you climb stairs at home:

Remember - tick one box only

- without any help? ☐
- with someone carrying your frame? ☐
- with someone encouraging you? ☐
- with physical help? ☐
- not at all? ☐
- don’t have stairs? ☐

Dressing

Do you get dressed:

Remember - tick one box only

- without any help? ☐
- just with help with buttons? ☐
- with someone helping you most of the time? ☐

Mobility

Do you walk indoors:

Remember - tick one box only

- without any help apart from a frame? ☐
- with one person watching over you? ☐
- with one person helping you? ☐
- with more than one person helping? ☐
- not at all? ☐
- Or do you use a wheelchair independently? (e.g. round corners) ☐

Transfer

Do you move from bed to chair:

Remember - tick one box only

- on your own? ☐
- with a little help from one person? ☐
- with a lot of help from one or more people? ☐
- not at all? ☐

For peer review only

Feeding

Do you eat food:	without any help?	☐
	with help cutting food or spreading butter?	☐
Remember - tick one box only	with more help?	☐

Toilet use

Do you use a toilet or commode:	without any help?	☐
	with some help but can do something?	☐
Remember - tick one box only	with quite a lot of help?	☐

Grooming

Do you brush your hair and teeth	without help?	☐
Wash your face and shave:	with help?	☐
Remember - tick one box only		

Bladder

Are you incontinent of urine?	never	☐
	less than once a week	☐
Remember - tick one box only	less than once a day	☐
	more often	☐
	Or do you have a catheter managed for you	☐

Bowels

Do you soil yourself?	never	☐
	Occasional accident	☐
Remember - tick one box only	all the time	☐
	or do you need someone to give you an enema?	☐

For peer review only

UNIVERSITY OF CAMBRIDGE

Queen Mary University of London

Help with taking tablets after stroke

FAMILY MEMBER/FRIEND OR PRIVATE CARER COPY

For peer review only

Many people with stroke need help with taking their medicines. We would like to ask you few questions about the help you might be offering to your family member/friend/ patient with stroke with taking medicines.

Relation with your family member/friend with stroke

Are you:

Remember - tick one box only

- partner ☐
- son or daughter ☐
- friend ☐
- carer from an agency ☐
- other ☐

if other, please specify

How many different types of medicines does your family member/friend/patient with stroke take in one day? ☐
Write the number or an approximation. Please count all medications including eye drops, injections etc.

How old is your family member/friend/patient with stroke? (years) ☐

How many years ago was your family member/friend/patient's stroke? ☐

What is your family member/friend/patient with stroke sex? M ☐ F ☐

For each question below, please tick the box that best describes the help needed by your family member/friend/patient with stroke with taking medicines <u>in the last month.</u>

1. **Is somebody helping your family member/friend/patient with stroke with prescriptions and collection of his/her medicines?**

   - all the time ☐
   - often ☐
   - sometimes ☐
   - rarely ☐
   - never ☐

Do you feel your family member/friend/patient with stroke needs more help with prescriptions and collection of his/her medicines?

Yes ☐ **No** ☐

Version 1.0 010316

☐☐.☐☐☐

2. Is somebody helping your family member/friend/patient with stroke getting the medicines out of the box, bottle or blister pack?

- all the time ☐
- often ☐
- sometimes ☐
- rarely ☐
- never ☐

Do you feel your family member/friend/patient with stroke needs more help with getting the medicines out of the box, bottle, or blister pack?

Yes ☐ **No** ☐

3. Is somebody helping your family member/friend/patient with stroke with reminding when is time to take his/her medicine?

- all the time ☐
- often ☐
- sometimes ☐
- rarely ☐
- never ☐

Do you feel your family member/friend/patient with stroke needs more help with reminding when is time to take his/her medicine?

Yes ☐ **No** ☐

4. Is somebody helping your family member/friend/patient with stroke with swallowing his/her medicine? For example by giving a drink.

- all the time ☐
- often ☐
- sometimes ☐
- rarely ☐
- never ☐

Do you feel you your family member/friend/patient with stroke need more help with swallowing his/her medicine?

Yes ☐ **No** ☐

5. Is somebody helping your family member/friend/patient with stroke with checking that he/she has taken his/her medicines?

- all the time ☐
- often ☐
- sometimes ☐
- rarely ☐
- never ☐

Do you feel your family member/friend/patient with stroke needs more help with checking that he/she has taken his/her medicine?

Yes ☐ **No** ☐

Version 1.0 010316

For peer review only

☐☐.☐☐☐

Missing medicines

Thinking of the last 30 days, how often did your family member/friend/patient with stroke miss taking his/her regular medicines?

Remember - tick one box only

- all the time ☐
- often ☐
- sometimes ☐
- rarely ☐
- never ☐

Barthel Questionnaire

These are some questions about the ability of your family member/friend/patient with stroke to look after him/herself.

Please answer them all.

Please fill this questionnaire even if you are not regularly caring for your family member/friend/patient with stroke, trying to answer questions in the way you think most accurately describes the disability of your family member/friend/patient with stroke.

Tick one box in each section.

Bathing

In the bath or shower do you:

Remember - tick one box only

- manage on your own? ☐
- need help getting in and out? ☐
- need other help? ☐
- never have a bath or shower? ☐
- need to be washed in bed? ☐

Stairs

Do you climb stairs at home:

Remember - tick one box only

- without any help? ☐
- with someone carrying your frame? ☐
- with someone encouraging you? ☐
- with physical help? ☐
- not at all? ☐
- don't have stairs? ☐

Dressing

Do you get dressed:

Remember - tick one box only

- without any help? ☐
- just with help with buttons? ☐
- with someone helping you most of the time? ☐

Mobility

Do you walk indoors:

Remember - tick one box only

- without any help apart from a frame? ☐
- with one person watching over you? ☐
- with one person helping you? ☐

Version 1.0 010316

For peer review only

	with more than one person helping?
	not at all?
	Or do you use a wheelchair independently? (e.g. round corners)

Transfer

Do you move from bed to chair:	on your own?
	with a little help from one person?
Remember - tick one box only	with a lot of help from one or more people?
	not at all?

Feeding

Do you eat food:	without any help?
	with help cutting food or spreading butter?
Remember - tick one box only	with more help?

Toilet use

Do you use a toilet or commode:	without any help?
	with some help but can do something?
Remember - tick one box only	with quite a lot of help?

Grooming

Do you brush your hair and teeth	without help?
Wash your face and shave:	with help?
Remember - tick one box only	

Bladder

Are you incontinent of urine?	never
	less than once a week
Remember - tick one box only	less than once a day
	more often
	Or do you have a catheter managed for you

Bowels

Do you soil yourself?	never
	Occasional accident
Remember - tick one box only	all the time
	or do you need someone to give you an enema?

For peer review only

Version 1.0 010316

STROBE 2007 (v4) Statement—Checklist of items that should be included in reports of *cross-sectional studies*

Section/Topic	Item #	Recommendation	Reported on page #
**Title and abstract**	1	(*a*) Indicate the study's design with a commonly used term in the title or the abstract	2
		(*b*) Provide in the abstract an informative and balanced summary of what was done and what was found	2
**Introduction**			
Background/rationale	2	Explain the scientific background and rationale for the investigation being reported	4-5
Objectives	3	State specific objectives, including any pre-specified hypotheses	5
**Methods**			
Study design	4	Present key elements of study design early in the paper	5-6
Setting	5	Describe the setting, locations, and relevant dates, including periods of recruitment, exposure, follow-up, and data collection	6
Participants	6	(*a*) Give the eligibility criteria, and the sources and methods of selection of participants	6-7
Variables	7	Clearly define all outcomes, exposures, predictors, potential confounders, and effect modifiers. Give diagnostic criteria, if applicable	9
Data sources/ measurement	8*	For each variable of interest, give sources of data and details of methods of assessment (measurement). Describe comparability of assessment methods if there is more than one group	8
Bias	9	Describe any efforts to address potential sources of bias	-
Study size	10	Explain how the study size was arrived at	6
Quantitative variables	11	Explain how quantitative variables were handled in the analyses. If applicable, describe which groupings were chosen and why	8-9
Statistical methods	12	(*a*) Describe all statistical methods, including those used to control for confounding	8-9
		(*b*) Describe any methods used to examine subgroups and interactions	8-9
		(*c*) Explain how missing data were addressed	-
		(*d*) If applicable, describe analytical methods taking account of sampling strategy	-
		(*e*) Describe any sensitivity analyses	-

For peer review only

**Results**			
Participants	13*	(a) Report numbers of individuals at each stage of study—eg numbers potentially eligible, examined for eligibility, confirmed eligible, included in the study, completing follow-up, and analysed	10
		(b) Give reasons for non-participation at each stage	-
		(c) Consider use of a flow diagram	-
Descriptive data	14*	(a) Give characteristics of study participants (eg demographic, clinical, social) and information on exposures and potential confounders	10
		(b) Indicate number of participants with missing data for each variable of interest	-
Outcome data	15*	Report numbers of outcome events or summary measures	
Main results	16	(*a*) Give unadjusted estimates and, if applicable, confounder-adjusted estimates and their precision (eg, 95% confidence interval). Make clear which confounders were adjusted for and why they were included	11-12
		(*b*) Report category boundaries when continuous variables were categorized	-
		(*c*) If relevant, consider translating estimates of relative risk into absolute risk for a meaningful time period	-
Other analyses	17	Report other analyses done—eg analyses of subgroups and interactions, and sensitivity analyses	-
**Discussion**			
Key results	18	Summarise key results with reference to study objectives	12-13
Limitations	19	Discuss limitations of the study, taking into account sources of potential bias or imprecision. Discuss both direction and magnitude of any potential bias	13-14
Interpretation	20	Give a cautious overall interpretation of results considering objectives, limitations, multiplicity of analyses, results from similar studies, and other relevant evidence	14-16
Generalisability	21	Discuss the generalisability (external validity) of the study results	17
**Other information**			
Funding	22	Give the source of funding and the role of the funders for the present study and, if applicable, for the original study on which the present article is based	3

*Give information separately for cases and controls in case-control studies and, if applicable, for exposed and unexposed groups in cohort and cross-sectional studies.

Note: An Explanation and Elaboration article discusses each checklist item and gives methodological background and published examples of transparent reporting. The STROBE checklist is best used in conjunction with this article (freely available on the Web sites of PLoS Medicine at http://www.plosmedicine.org/, Annals of Internal Medicine at http://www.annals.org/, and Epidemiology at http://www.epidem.com/). Information on the STROBE Initiative is available at www.strobe-statement.org.

For peer review only
